# Diabetic Foot Talk-Time: framework for effective communication in diabetic foot management

**DOI:** 10.3389/fcdhc.2025.1590570

**Published:** 2025-06-23

**Authors:** Lavinia Cacciatore, Marco Meloni

**Affiliations:** ^1^ Master of Diabetic Podopathy, University of Rome Tor Vergata, Rome, Italy; ^2^ Diabetic Foot Unit, Department of Internal Medicine, University of Rome “Tor Vergata”, Rome, Italy

**Keywords:** diabetic foot, effective communication, prevention, information and comm. technologies (ICT), educational therapy, interdisciplinarity

## Abstract

**Introduction:**

Diabetic foot syndrome is a prevalent and costly chronic complication of diabetes mellitus, linked to high mortality rates and significant psychological and social burdens. These challenges exacerbate the disease’s impact and can hinder the ability of healthcare professionals to effectively connect with patients. Therapeutic education and effective communication can play crucial roles in fostering patient empowerment and adherence to care, which can help reduce frustration for both patients and caregivers. However, there is currently a lack of specific guidelines to direct healthcare professionals in diabetic foot care. This study employs a mixed-methods approach, integrating a systematic literature review and a cross-sectional survey, to evaluate existing communication strategies and develop a structured digital framework aimed at improving diabetic foot care. The research focuses on reviewing recent literature (from the past five years) on effective communication and therapeutic education in the prevention and management of Diabetic Foot Syndrome. Additionally, it includes an analysis of existing manuals on communication strategies and a descriptive survey to assess professional–patient and interprofessional communication challenges, identify areas for improvement, and measure levels of awareness among diabetic patients.

**Materials and methods:**

The literature review was conducted using the PICO method on the Medline database through PubMed, yielding 273 articles, of which eight were selected for in-depth analysis. A survey, conducted over four months, included 165 participants divided into professional and diabetic groups, each receiving targeted questionnaires.

**Results:**

The analysis of selected articles and communication manuals highlighted key themes aligned with the study’s objectives. Findings emphasized that self-management, effective communication, professional training in therapeutic education, and the use of information and communication technologies (ICT) are essential to improving patient adherence to diabetic foot care and optimizing therapeutic outcomes. Survey results revealed that a large proportion of diabetic patients reported either not receiving information on diabetic foot syndrome from healthcare professionals or only receiving it post-complication, leading many to seek information online. Both professionals and patients acknowledged that online resources enhance adherence to care.

**Discussion:**

The study underscores the need for reliable, accessible resources, including multimedia support for active health education aimed at both healthcare professionals and diabetic patients at risk of foot complications. Based on these findings, a prototype framework was developed—a web platform designed to support professionals and diabetic patients with features such as daily podiatric routines, alert systems, instructional images, and practical examples to integrate into clinical practice. Additionally, the platform includes a community space for feedback and interprofessional communication. The vision is to develop a mobile application a “virtual network of connection” designed to enhance the training of healthcare professionals and improve the care of diabetics with at-risk feet. This online framework could serve as a valuable tool to motivate and guide both professionals and patients along a path of effective prevention and care. By integrating into a secure web-based health network, it aims to provide accessible, reliable resources for better management of diabetic foot health.

## 1 Introduction

Managing diabetic foot is a multifaceted challenge, extending beyond clinical concerns to encompass significant psychological and social dimensions. Diabetes patients face not only the physical complications of the disease but also a substantial psychological burden that diminishes their quality of life and impairs treatment adherence. This emotional strain often leads to disengagement from healthcare professionals, complicating effective care (1, 2).

Health professionals play a pivotal role in enhancing patient quality of life and mitigating the disease’s impact (3). This can be achieved through effective communication and targeted therapeutic education. A primary challenge today is empowering caregivers, fostering self-efficacy, and promoting self-management. Studies show that communication breakdowns have a significant impact on treatment adherence and clinical outcomes. For example, diabetes patients often receive a lack of clear guidance on self-care routines, leading them to seek out unreliable online information sources. Additionally, healthcare providers cite difficulties in effectively conveying the severity of diabetic foot complications, resulting in delayed responses from patients. The Health Professional and the interdisciplinary Diabetic Foot Team can significantly impact patients’ involvement in self-care by offering guidance and support (3, 4).

Currently, no specific guidelines exist for communication within the context of diabetic foot care, highlighting the need for a conceptual framework that equips healthcare professionals with tools to communicate effectively with patients. This study identifies specific weaknesses in current communication strategies and presents a new framework that leverages digital technology for professional-patient engagement. Such a framework is crucial for optimizing syndrome management and improving patient quality of life (5). Designed to be fully online, this framework leverages the growing trend of technology use in healthcare (6, 7). The advantage of an online format lies in its accessibility, allowing patients to engage with the material independently. As interest in paper-based resources wanes, this digital tool provides an invaluable multimedia platform for modern health education on diabetic foot care (7).

This tool capitalizes on technological advancements in healthcare, prioritizing accessibility and usability (8). Its goal is to facilitate productive communication between diabetes patients and healthcare professionals, promote patient self-management, and enhance collaboration within interdisciplinary team.

It aims to bridge gaps that hinder active care adherence by encouraging foot self-monitoring, fostering patient empowerment, and motivating safe behavioral practices (6, 7). Additionally, it serves as a guide for professional dialogue, reinforcing the importance of team collaboration in diabetic foot management and integrating health professionals through proactive behavioral patterns.

### 1.1 Goals

The study aims to identify key strategies for improving care adherence in patients with diabetic foot by optimizing therapeutic outcomes and enhancing communication skills among healthcare professionals within the interdisciplinary team.

The framework’s implementation unfolds in two phases. The first phase, central to the project, involved a literature review focused on key topics such as effective communication, therapeutic education, and patient engagement in diabetic foot prevention and management. Scientific articles addressing communication between patients with at-risk feet and healthcare providers, as well as the involvement of diabetics in care planning, were reviewed. Furthermore, manuals, guides, and communication models from other medical fields, including rehabilitation-orthopedic and oncology disciplines, were examined to assess how healthcare professionals approach patients with acute and chronic conditions. This analysis identified three key manuals.

An experimental study was conducted as a pivotal phase of the research using a descriptive survey questionnaire model. The study aimed to:

- Investigate the approach of health professionals and diabetic practitioners to disease management
- Explore the role of effective communication in diabetic foot management;
- Examine the goals, obstacles, and strategies of health professionals in establishing effective communication with diabetic patients and the interdisciplinary diabetic foot team, regardless of whether these professionals are present
- Investigate the level of awareness among diabetic patients.

## 2 Methodology

### 2.1 Literature review

The research question focused on the effectiveness of communication between patients with diabetic foot syndrome and health professionals, along with the role of therapeutic education and patient involvement in the treatment plan. The PICO method (P: population/patient; I: intervention/indicator; C: comparator/control; O: outcome) was employed for the literature search in the Medline database through PubMed, without any time limits. Search terms related to the target issue of the project— “diabetic foot”—were paired with intervention-related terms, including “effective communication skills,” “educational therapy,” and “patient engagement,” and outcome-related terms, such as “diabetic foot prevention” and “self-care for diabetic foot.” These terms were combined using Boolean operators (AND, OR).

**Figure 1:**
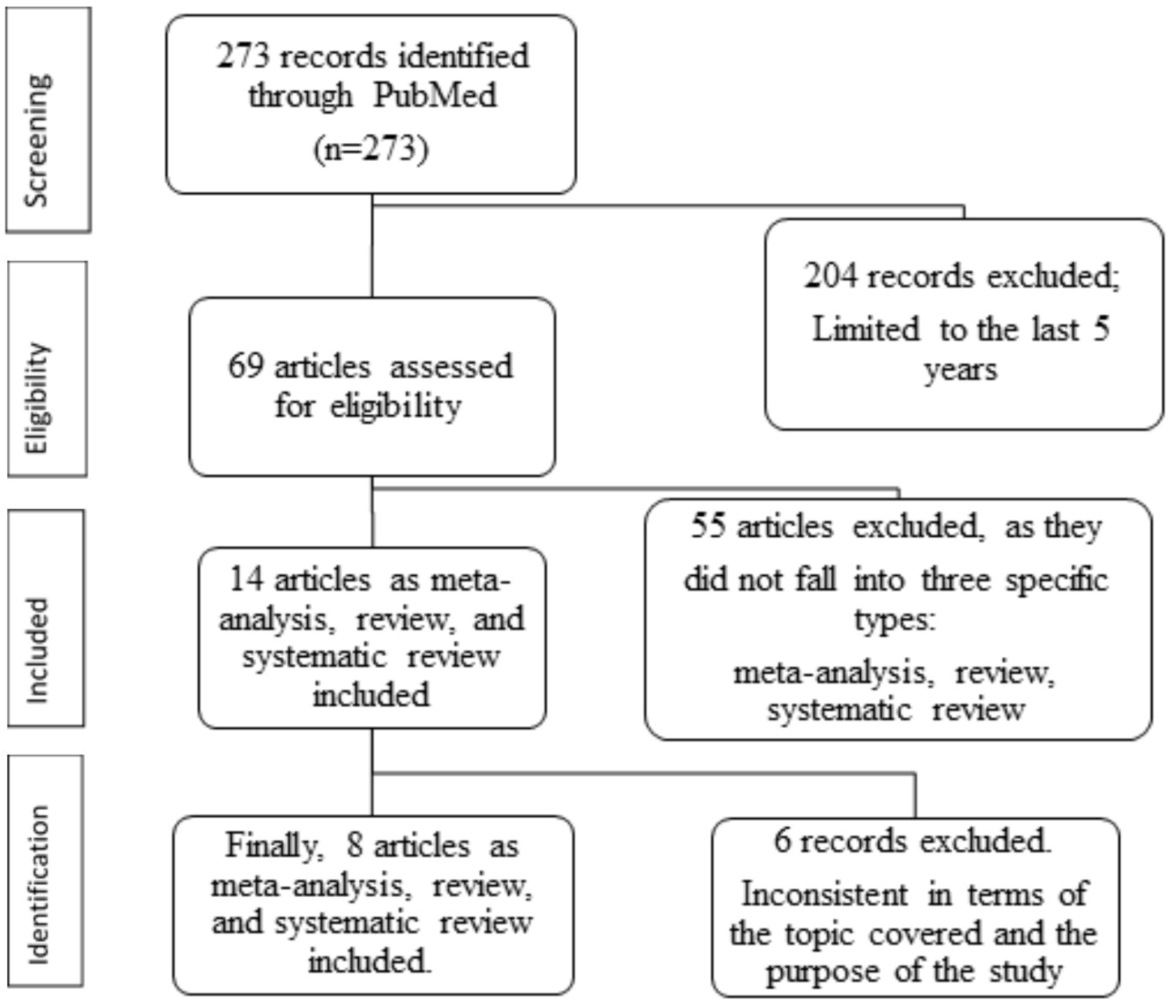
Flowchart of the study design. Illustrates the flowchart of the study design, detailing the systematic process of article selection.

The search resulted in 273 scientific articles. After an initial screening, articles published beyond the last 5 years were excluded, leaving 69 relevant articles. Next, articles were filtered to include only meta-analyses, reviews, or systematic reviews, resulting in 14 articles. Of these, 6 were excluded for being inconsistent with the study’s subject and objectives. The final selection included 8 eligible articles (**Figure 1**), which were summarized in **Table 1**. These studies were chosen based on their focus on patient education, interprofessional communication, and digital interventions, which are critical components of the proposed framework. A PRISMA flowchart has been included to enhance methodological transparency.

**Table 1:** Characteristics of the included studies.

N°	Title	Authors	Year	Source
8	Perceptions and experiences of diabetic foot ulceration and foot care in people with diabetes: A qualitative meta-synthesis.	Coffey L,Mahon C, Gallagher P (9).	2019	PubMed International Wound Journal
9	Use of Information Communication Technology Tools in Diabetic Foot Ulcer Prevention Programs: A Scoping Review.	Obilor HN, Achore M, Woo K.Can J (10)	2022	PubMed Canadian Journal of Diabetes
10	Overcoming barriers to self-management: The person-centered diabetes foot behavior agreement.	Bullen B,Young M, McArdle C, Ellis M (11)	2019	PubMed The Foot
11	Prevention of foot ulcers in the at-risk patient with diabetes: a systematic review.	Van Netten JJ, Raspovic A, Lavery LA, Monteiro-Soares M, Rasmussen A, Sacco ICN, Bus SA (12).	2020	PubMed Diabetes Metabolism Research and Review
12	Cost-effectiveness of Interventions to Manage Diabetes: Has the Evidence Changed Since 2008?	Siegel KR, Ali MK, Zhou X, Ng BP, Jawanda S, Proia K, Zhang X, Gregg EW, Albrigh AL, Zhang P (13)	2020	PubMed Diabetes care ADA
13	Behavior change approaches for individuals with diabetes to improve foot self-management: a scoping review.	Paton J, Abey S, Hendy P, Williams J, Collings R, Callaghan L.J (14)	2021	PubMed Journal of Foot and Ankle Research
14	Influence of Health Education on Podiatric Knowledge, Self-care, and Conditions in Adults With Diabetes Mellitus: A Systematic Review.	Fernández-León P, Palomo-Toucedo IC, Carvajal-Moreno L, Domínguez-Maldonado G, Sánchez-Sánchez S, Reina-Bueno M (15)	2022	PubMed Advances in Skin and Wound care
15	Treatment of modifiable risk factors for foot ulceration in persons with diabetes: a systematic review.	Van Netten JJ, Sacco ICN, Lavery LA, Monteiro-Soares M, Rasmussen A, Raspovic A, Bus S)A (16).	2020	PubMed Diabetes Metabolism Research and Review

This table provides an overview of the studies included in the review.

The reviews highlighted several critical factors in the prevention and management of diabetic foot ulcers (DFU).

Two reviews [see (9, 10)] emphasized the importance of adequate education and training—both traditional and through modern technologies like Information and Communication Technologies (ICT)—to help patients understand the importance of self-care and preventive practices. Another two reviews [see (15, 16)] identified education and knowledge provided by healthcare professionals as key motivators for patient engagement in foot self-care.

Regular self-management practices, such as daily foot inspections and wearing preventive footwear, were identified as crucial for reducing the risk of ulceration [see (16)]. Two reviews [see (9, 14)] stressed that consistent, specific practices are essential for preventing complications, improving symptoms, and promoting long-term foot health, suggesting that self-management should be central to prevention programs.

Three reviews [see (9, 11, 14)] revealed several barriers to effective self-management, such as inadequate communication from healthcare professionals and a lack of understanding about the condition. Improving communication and adopting a person-centered approach were found to help overcome these obstacles.

Two reviews [see (10, 13)] demonstrated that ICT can enhance communication, support remote monitoring, and provide effective educational tools, ultimately reducing DFU recurrences and improving adherence to self-management practices. This approach is also cost-effective, as it reduces long-term healthcare costs associated with diabetic foot complications.

Lastly, three studies [see (9, 14, 16)] highlighted the benefits of a multidisciplinary approach, in which specialists collaborate to provide coordinated care and improve quality of life by preventing complications.

The studies emphasize the importance of a holistic approach that integrates education, self-management, technology, effective communication, and multidisciplinary involvement to enhance the autonomy and confidence of diabetics in managing their condition.

#### 2.1.1 Analysis manuals, guides, or models of effective communication in the literature

In addition, manuals, guides, and models of effective communication with caregivers—covering diseases and disciplines beyond diabetic foot care, including rehabilitation, orthopedics, and oncology—were analyzed to assess the approach of healthcare professionals toward patients with both acute and chronic conditions.

Three key manuals emerged from this analysis:

- *Manual of Communication Assessment in Rehabilitation* (ISTISAN Reports 13/1, Istituto Superiore della Sanità)
- *Physician-Patient Communication and Between Health Professionals* (Module II, “Communication and Professional Performance: Methods and Tools,” Ministry of Health)
- *Therapeutic Education for the Person with Diabetes* (Chapter 24, *Handbook for Practitioners: “Educating for Health and Care”*).

All manuals emphasize the importance of clear, empathetic, and patient-centred communication to enhance understanding and collaboration. They also highlight the necessity of continuous therapeutic education for patients and regular training for healthcare professionals.

These resources have been reviewed and integrated into the development of the online framework, providing insights into successful strategies that can be applied to diabetic foot care.

## 3 Methodology

### 3.1 Experimental study with a descriptive survey questionnaire model

An experimental study, based on a review of scientific literature, was conducted over a 4-month period (May to August 2023). It involved distributing two questionnaires via Google Forms—one for healthcare professionals and the other for diabetic patients.

#### 3.1.1 Questionnaire design: mixed (qualitative and quantitative) and quantitative approach

The questionnaire design employed a mixed-methods approach to capture both qualitative and quantitative data from healthcare professionals, while a quantitative approach was used for the diabetic patient survey to streamline analysis.

The healthcare professionals’ questionnaire was developed with the support of faculty from the Master’s program in Diabetic Podopathy at the University of Rome Tor Vergata. It was then disseminated to professionals across Italy through various channels (WhatsApp, email).

The diabetic patient questionnaire, based on the 2001 GISED questionnaire on Diabetic Awareness, Diabetic Foot, and Quality of Life, was adapted for this project and tailored to the local health context of Licata, a town in the province of Agrigento, Sicily. It also incorporated insights from the permanent solidarity screenings for diabetic foot care conducted at San Giacomo D’Altopasso Hospital, which lacks a dedicated diabetic foot clinic. The screenings were carried out in collaboration with Lions Club Licata and the Tribunal for Patients’ Rights, providing free services to diabetics in socioeconomically disadvantaged conditions. These screenings revealed a significant lack of awareness regarding complications related to diabetic foot conditions.

To assess the local diabetic population’s awareness, the questionnaire was distributed over four months to individuals attending a private podiatry practice and those seeking free screenings. This effort aimed to gauge the level of knowledge among diabetics, which could reflect broader trends in regional and national healthcare systems where the response to emerging conditions such as diabetic foot syndrome is often insufficient.

#### 3.1.2 Sending questionnaire

Questionnaires were distributed to a diverse group of professionals specializing in various fields and diabetics with varying degrees of foot involvement. For professionals, dissemination occurred through links shared on messaging platforms (WhatsApp, Messenger), the Secretariat of the Master’s Program in Diabetic Podopathy at the University of Rome Tor Vergata, collaborations with professors and students of the program, the Territorial Boards of Podiatrists from various regions across Italy, and the Sicilian Podiatry Group via WhatsApp. The questionnaire for diabetics was promoted through the Solidarity Screening of the Diabetic Foot at the OP of Licata and among patients from a private podiatry practice in Licata. After a documented diabetes diagnosis, the questionnaire was sent via link. For patients who had difficulty using digital tools, a paper version of the questionnaire was provided, with data later entered into the platform (17).

#### 3.1.3 Sample of participants: health professionals and diabetics

The sample population was selected to include a variety of professionals involved in diabetic foot care and patients with diabetes.

A random sampling method was used, with 165 participants in total: 85 health professionals and 80 diabetics. This sample size is considered sufficient to gather diverse perspectives and to explore the dynamics of effective communication in this context.

### 3.2 Selection criteria

The 85 health professionals were selected based on their specialization in diabetes and diabetic foot management, regardless of their participation in an interdisciplinary team. Diabetic participants were chosen based on their documented diagnosis and willingness to take part in the study. The sample included individuals of various ages and both genders, without distinction.

Informed consent was obtained from both professionals and patients prior to participation.

#### 3.2.1 Health professionals as champion

The health professionals group includes

- Endocrinologist/Diabetologist
- Cardiologist
- General surgeon
- Plastic surgeon
- General practitioner
- Podiatrist
- Nurse
- Physiotherapist
- Orthopedic technician
- Psychologist
- Health educator
- The professionals who participated in the study belonged to different work backgrounds such as:
- Hospitals: departments of diabetes, endocrinology, internal medicine, surgery, orthopedics, and emergency room
- Dedicated diabetic foot outpatient clinics or referral centers
- Basic medical studies
- Private professional firms
- Territorial home care

The professionals also varied in terms of work experience, both in professional settings and in the duration of their experience with diabetic foot management.

#### 3.2.2 Sample patients with diabetic foot

The diabetic sample consisted of 80 individuals from diverse cultural, social, and socioeconomic backgrounds, representing a range of clinical characteristics, including both type 1 and type 2 diabetes, differing in:

- duration of diabetes,
- glycemic control levels
- levels of education
- severity of foot condition
- diabetic foot complications
- And multiple levels of involvement:
- Diabetics who are approaching foot treatment for the first time
- With an active diabetic foot
- With diabetic foot at risk but without current symptoms
- With chronic diabetic foot or with past experience of complications
- Patients who have undergone amputations due to the diabetic foot

The heterogeneity in the sample ensures that the manual on effective communication is adaptable to the needs of a wide range of health professionals and individuals with diabetic foot syndrome.

### 3.3 Data collection tools: description of questionnaires

#### 3.3.1 Questionnaire for healthcare professionals

The questionnaire for health professionals included both open-ended and multiple-choice questions to assess their experiences, challenges, expectations, and objective communication skills. Open-ended questions allowed participants to express their views freely, facilitating the discovery of new, unconsidered themes. This approach provided valuable qualitative insights into the relationship between diabetics and professionals, as well as substantial quantitative data on current communication skills and areas for improvement. The survey’s findings are intended to inform future training programs focused on therapeutic education and effective communication.

To streamline data collection, the questionnaire was divided into seven thematic sections.

(see in the appendix)

#### 3.3.2 Questionnaire for diabetic patients

The survey uses a quantitative approach with multiple-response items, enabling structured data collection that facilitates analysis. Its objectives include assessing: the perception of communication, patient satisfaction, the ability to self-manage and self-monitor, the quality of information provided by healthcare professionals, the impact of current communication strategies, the role of caregivers in daily life, and opportunities for improving healthcare. Similar to the previous questionnaire, this patient survey is organized into sections corresponding to the topics covered.

(**Supplementary Tables**).

## 4 Results

### 4.1 Quantitative analysis of data from the questionnaire dedicated to health professionals

**Section 1 Information about the practitioner**

The professional sample included individuals from a range of disciplines, such as:

- Endocrinologist/Diabetologist
- Cardiologist
- General surgeon
- Orthopedic surgeon
- Plastic surgeon
- General practitioner
- Podiatrist
- Nurse
- Physiotherapist
- Orthopedic technician
- Psychologist
- Health educator

**Figure 2:**
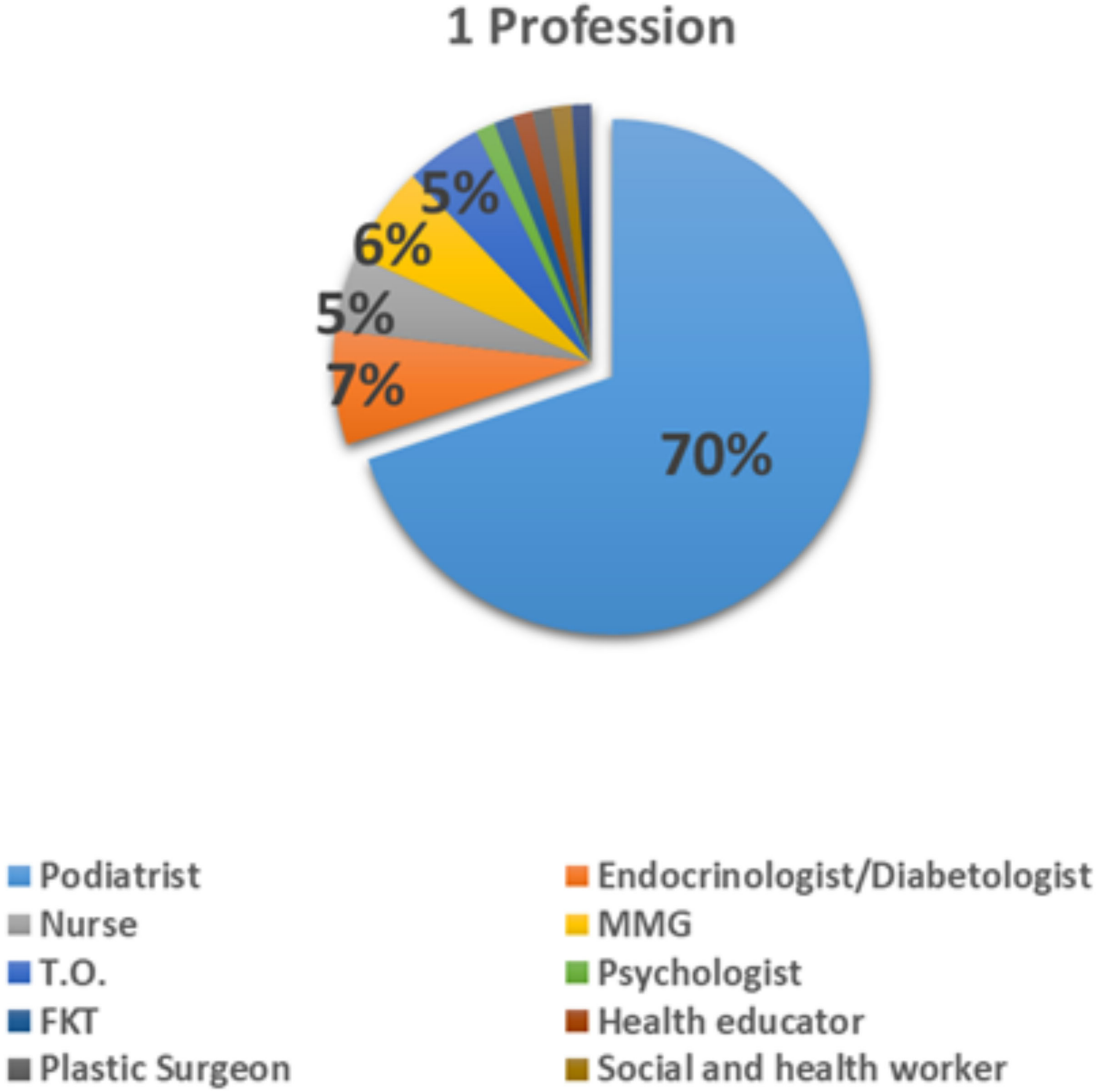
Question 1 of the questionnaire for professionals: professional roles of healthcare practitioners.

Among all professions, 68.7% (the majority) were podiatrists (**Figure 2**).

The professionals who participated in the study belonged to different work backgrounds such as

- Hospitals: departments of diabetes, endocrinology, internal medicine, surgery, orthopedics, and emergency room
- Dedicated diabetic foot outpatient clinics or referral centers
• Basic medicine studies
• Private professional firms
• Territorial home care

**Figure 3:**
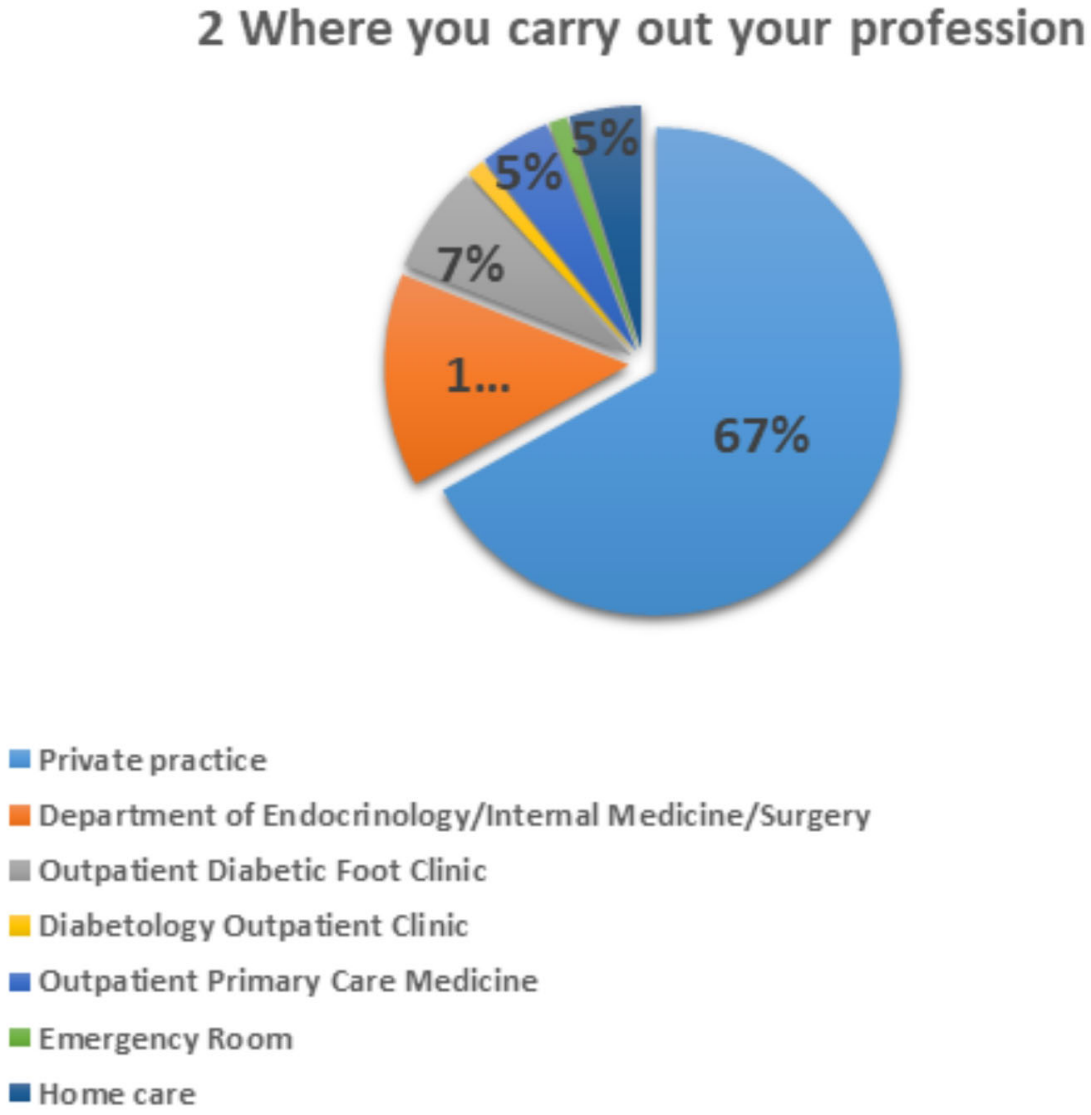
Question 2 of the questionnaire for professionals.

A total of 63% of professionals carried out their work in private professional practices (**Figure 3**).

Regarding the practitioner’s role within the team, 71.1% of respondents indicated they were part of the Interdisciplinary Diabetic Foot Team.

**Figure 4:**
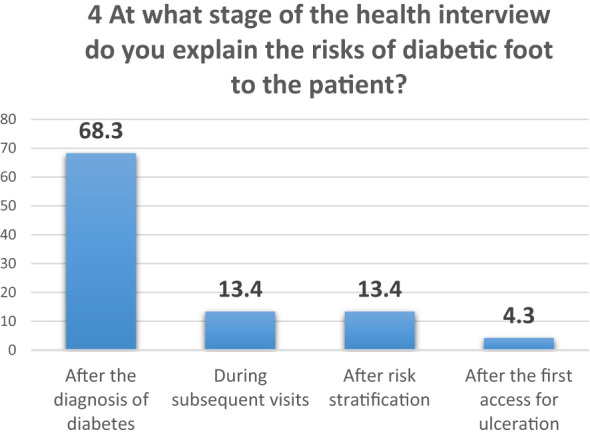
Question 4 of the questionnaire for professionals.

Analysis of the fourth question reveals a clear trend: 68.3% of healthcare professionals inform patients diagnosed with diabetic foot syndrome about the associated risks immediately after the diagnosis (**Figure 4**).

For the fifth question, 62.2% of professionals reported that they do not provide paper-based informational support to diabetic patients.

**Figure 5:**
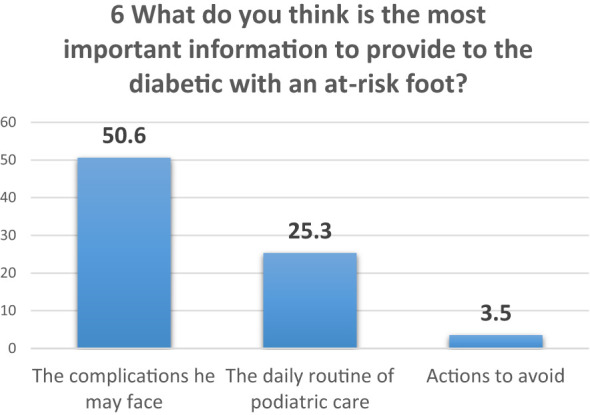
Question 6 of the questionnaire for professionals.

The sixth question assessed professionals’ views on the most critical information to share with diabetic patients at risk of foot complications. The majority of respondents (50.6%) identified the education of patients regarding potential lifelong complications as essential (**Figure 5**).

For question 7, there was an almost unanimous answer (90%), that is, the practice of educating the caregiver.

**Section 3 Expectations**

Section 3 of the survey explores healthcare professionals’ expectations regarding patients’ understanding of information and its application, based on follow-up feedback.

In question 8, 50% of professionals believed that diabetic patients did not fully understand the risks and complications associated with non-compliance to therapeutic education guidelines.

In contrast, question 9 revealed that 64% of healthcare professionals felt that most patients adhered to the advice provided during healthcare consultations.

**Section 4 Challenges and obstacles to communication**

Section 4 addresses the challenges and barriers in communication between professionals and diabetic patients.

In question 10, professionals were asked to rate the difficulty of communicating with diabetic patients on a scale from 1 (least difficult) to 10 (most difficult). A notable proportion reported high levels of difficulty, with ratings of 5 and above increasing significantly.

**Figure 6:**
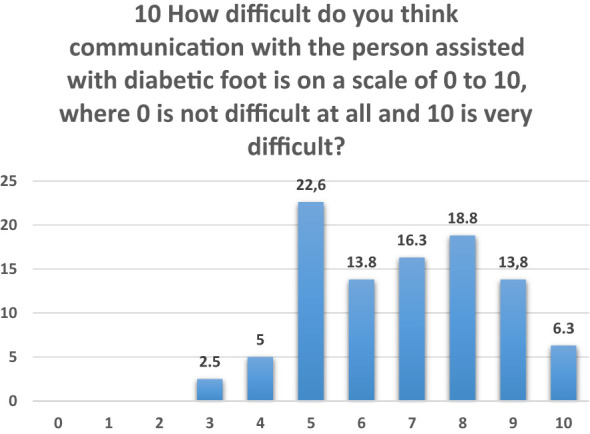
Question 10 of the questionnaire for professionals.

Among these, 22.6% of healthcare professionals rated the communication difficulty at level 5, followed by 18.8% at level 8, and 16.8% at level 7. Additionally, 13.8% of respondents indicated difficulties at levels 6 and 9 (**Figure 6**).

Section 5 focuses on strategies for improving communication. An overwhelming 80% of professionals agreed that increased adherence to diabetic foot care guidelines could be achieved through online consultations via a dedicated website.

Finally, the quantitative item in Section 7, titled “Suggestions and Improvements,” reveals that 98% of professionals believe it is essential to implement specific training courses to enhance communication among all individuals involved in diabetic foot care.

### 4.2 Qualitative analysis of themes emerging from the questionnaire dedicated to health professionals

The qualitative analysis of open-ended survey items from health professionals identified key topics that deepen our understanding of communication and therapeutic education in the critical clinical setting.

Each response was meticulously evaluated to identify, understand, and organize recurring patterns. Initially, the responses were transcribed and analyzed, then categorized into meaningful units—fragments of text encapsulating distinct concepts. These were grouped under codes, with similar themes organized into categories. Relationships between categories were explored to uncover connections and, when appropriate, subcategories were assigned.

### 4.3 Emerging themes and subcategories

In Section 4, Challenges and Barriers to Communication, question 8 invited professionals to reflect on the major obstacles they encounter when communicating with diabetic patients, offering nuanced insight into factors that may hinder effective care delivery.

The themes that emerged from participants’ responses, listed in descending order, highlight these barriers.

- Underestimation and dissipation of disease risks
- Educational level and socioeconomic-cultural barriers
- Rejection of the clinical condition
- Ineffective communication
- Poor compliance
- Emotional Factors (Fear)
- Absence of Caregiver
- Examining the responses to question 9, “What do you think the assisted person does not understand, to a greater extent, about his or her condition?”, 2 themes mainly emerge:
- Complications
- Prevention
- The severity and complexity of the pathology
- For question 10, which describes the aspects that the assisted person does not understand about what he or she is told during the health interview by the professional, stands out among all the questions:
- Prevention understood as:
  The observance and use of proper daily foot hygiene and inspection practices, what we might call “podo-daily-routine”
  The use of therapeutic footwear in primary and secondary prevention and unloading aid in the acute “off-loading” phase.
- Followed by
- Risks and complications.
- Continuing in the qualitative analysis, in section 5, “Strategies for improving communication,” in question 11, where professionals are asked how they think if it is possible to help the person assisted adhere to the treatment pathway, three themes stand out, in the order of prevalence:
- Effective communication
- Structured therapeutic education
- Continuous follow-ups
- Capillary diffusion
- Prevention
- Caregiver
- Team

Below, two separate sections were designed reserved for collaboration among health professionals and suggestions for improvement.

Section 6, “Challenges to Interdisciplinary Collaboration,” contains two questions. Question 13 asks practitioners about communication challenges with other professionals in the interdisciplinary Diabetic Foot Team. The responses, evaluated for key themes, reveal the primary barriers in this context, organized in descending order.

- Insufficient collaboration and structured communication
- Lack of awareness
- Lack of time
- In Question 14, which addresses communication with individuals outside the team, four distinct themes emerged from the responses.
- Lack of knowledge and contextual differences
- Reduced team awareness and professional silos
- Poor interprofessional communication

In the last section, “Suggestions and Improvements,” question 15 is related to the strategies that might be adopted to improve interprofessional communication. The suggestions put forward are varied:

- Specific and continuing education
- Interdisciplinary meetings
- Clear roles and responsibilities
- Adoption of standard protocols
- Interdisciplinary team

### 4.4 Quantitative analysis of data from the questionnaire dedicated to diabetic patients

The survey responses from diabetic patients offer valuable insight into their awareness of diabetic foot risks, perceptions of communication with healthcare professionals, satisfaction with educational resources, and capacity for self-management. This section summarizes the key quantitative findings from the patient questionnaire.

In Section 1, “Risk Awareness,” 70% of respondents indicated an awareness of the risks associated with diabetic foot complications. However, 30% lacked the necessary information to understand these risks.

The second item asks diabetics to assess their understanding of major complications related to diabetic foot syndrome. The responses revealed the following:

- 61% of caregivers understand the meaning of diabetic neuropathy.
- 54.4% understand the meaning of the term lower limb arteriopathy.
- 79.7% of respondents understand the meaning of ulcer in the context of the diabetic foot.

Section 2, “Communication and Education,” Item 5 explores the healthcare interview, asking patients whether health professionals explained the significance of diabetic foot complications. While 50% of respondents reported receiving this information, the other half either did not remember receiving it or were not provided with adequate knowledge.

**Figure 7:**
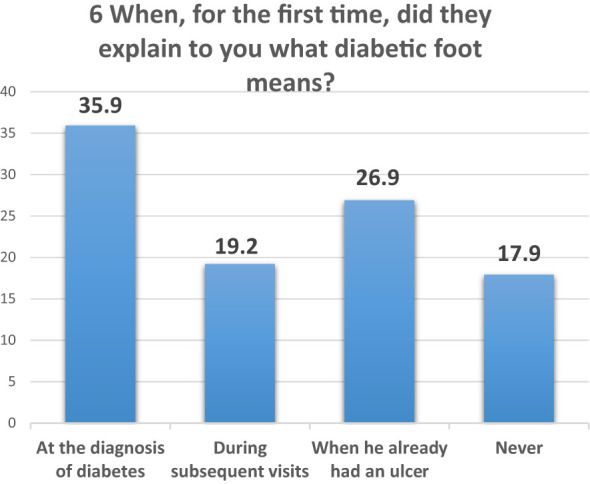
Question 6 of the questionnaire for patients.

Item 6 assesses when information about diabetic foot risks was communicated to patients (**Figure 7**):

- 35.9% were informed upon diagnosis of diabetes;
- 26.9% were informed after developing an ulcer;
- 19.2% received this information during follow-up visits;
- 17.9% reported never receiving information about diabetic foot risks.

In response to the follow-up question, “Did they explain to you how to manage your foot?”, 69.6% of participants answered affirmatively.

Regarding the completeness and satisfaction with the information provided by healthcare personnel on diabetic foot management (question 8), 58.2% of participants expressed satisfaction. However, 41.8% indicated dissatisfaction, highlighting gaps in communication during healthcare interactions.

An additional key finding in feedback from patients comes from responses to question 9. Fifty-seven percent of diabetics reported that the information they received about diabetic foot complications did not cause fear.

However, 43% of respondents expressed concern or fear.

In item 10, 70.5% of diabetics stated that they did not receive any printed educational materials after their healthcare consultation.

Continuing in the “Feedback and Satisfaction” section, 73% of respondents reported using Google to search for information about diabetic foot (question 11).

Despite this, while 73% sought online resources, only 64% felt they understood the information they found.

A notable finding comes from item 13, where 75% of diabetics reported no difficulty in managing their foot, and 78% believed they could follow diabetic foot care guidelines provided by healthcare professionals or found online with ease.

**Figure 8:**
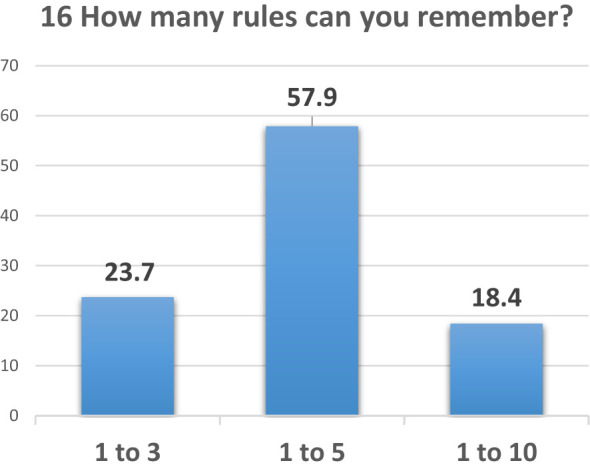
Question 16 of the questionnaire for patients.

In contrast, 71% found it easy to remember the foot care rules; however, when asked in question 16, “How many rules can you remember?”, only 58% of the diabetic population could recall 1 to 5 rules, 24% recalled 1 to 3 rules, and just 18% remembered up to 10 rules (**Figure 8**).

The majority of participants (70%) reported they could apply the recommendations from healthcare professionals or found online (question 17). Despite this, 30% of respondents struggled with implementation, though only 24% found it difficult.

In the “Directions for Improvement” section, 81% of participants (question 19) expressed that it would be easier to access foot care recommendations through their smartphones.

The final item of the survey reveals that 67% of diabetics receive support from a caregiver in their daily lives.

## 5 Discussion

### 5.1 Quantitative data analysis of questionnaires dedicated to health professionals and diabetics

The quantitative analysis of the data collected from questionnaires directed to both health professionals and diabetic patients provides valuable insights into relational dynamics, educational communication, and the management of diabetic foot syndrome. Both groups offer complementary perspectives, highlighting challenges, gaps, and potential improvements in clinical practices and preventive behaviors. Key findings, as discussed below, relate the responses from both groups, offering an overview of interactions and perceptions regarding diabetic foot management.

### 5.2 Participation and profile of respondents

Among health professionals, 68.7% of respondents were podiatrists, with 63% working in private practice. This finding is significant as it suggests that professional affiliation and private practice may encourage greater participation in studies focused on specific clinical issues like diabetic foot syndrome. On the patient side, the questionnaire reveals that 70% of respondents are aware of the risks associated with diabetic foot, indicating a solid foundational understanding of the condition. However, 30% of the population remains insufficiently informed, highlighting the need for enhanced education and awareness initiatives.

### 5.3 Communication and education

A key theme emerging from both questionnaires is communication and education. According to healthcare professionals, 62.2% do not provide paper support materials to diabetic patients, likely due to disinterest, logistical challenges, or time constraints. This finding is corroborated by patients, with 70.5% reporting they did not receive any support materials after consultations. The lack of paper resources may hinder patients’ ability to review and retain information from visits, limiting their capacity to remember and follow foot care recommendations.

### 5.4 Perception of understanding and storage of information

Another notable point of divergence is the perceived understanding of information and patients’ ability to retain recommendations. Approximately 50% of professionals believe patients do not fully comprehend the risks and complications of diabetic foot care. Conversely, 64% of diabetic patients claim to follow the provided recommendations. This suggests a discrepancy between patients’ perceived understanding and their actual comprehension of the risks involved. Furthermore, only 58% of patients can recall between 1 and 5 prevention rules, indicating significant gaps in information retention.

### 5.5 Role of the caregiver and importance of caregiver education

A positive development is the recognition by healthcare professionals of the critical role caregivers play in managing diabetic foot care. Ninety percent of professionals report educating caregivers on foot hygiene, inspection, and care practices, as well as recognizing warning signs that could prevent serious complications such as ulcers and infections. This is echoed by the patient survey, where 67% of diabetics report receiving caregiver support in their daily routines. This highlights the essential role caregivers play in coordinating care and addressing daily challenges, underscoring the need for continuous, targeted training for caregivers, in line with the IWGDF 2023 guidelines.

### 5.6 Use of technology and online resources

Both healthcare professionals and diabetic patients show growing interest in utilizing technology and online resources for education and support. Eighty percent of professionals believe a dedicated website could enhance adherence to diabetic foot care recommendations. This is supported by 73% of patients, who actively seek information online about diabetic foot care, and 81% who prefer to access recommendations via their smartphones. However, misinformation remains a concern, highlighting the need for a validated digital framework. Professional training programs also lack modules dedicated to diabetic foot communication, making it necessary to include structured communication guidelines within medical curricula. Developing a QR code linking to an online platform could significantly improve access to information, promoting greater awareness and care for diabetic foot health.

### 5.7 Satisfaction and feedback on health interviews

Satisfaction with the information provided is mixed. Among patients, 58.2% were satisfied with the completeness of the information received on diabetic foot care, while 41.8% felt there were communication gaps. This suggests that, despite ongoing educational efforts, there is room for improvement in the quality and clarity of the information conveyed during consultations. To enhance patient outcomes, it is essential to adopt clearer and more accessible communication strategies, ensuring that all individuals receive and fully understand the guidance necessary to manage their condition effectively.

### 5.8 Awareness of risk in significant subgroups

**Figure 9:**
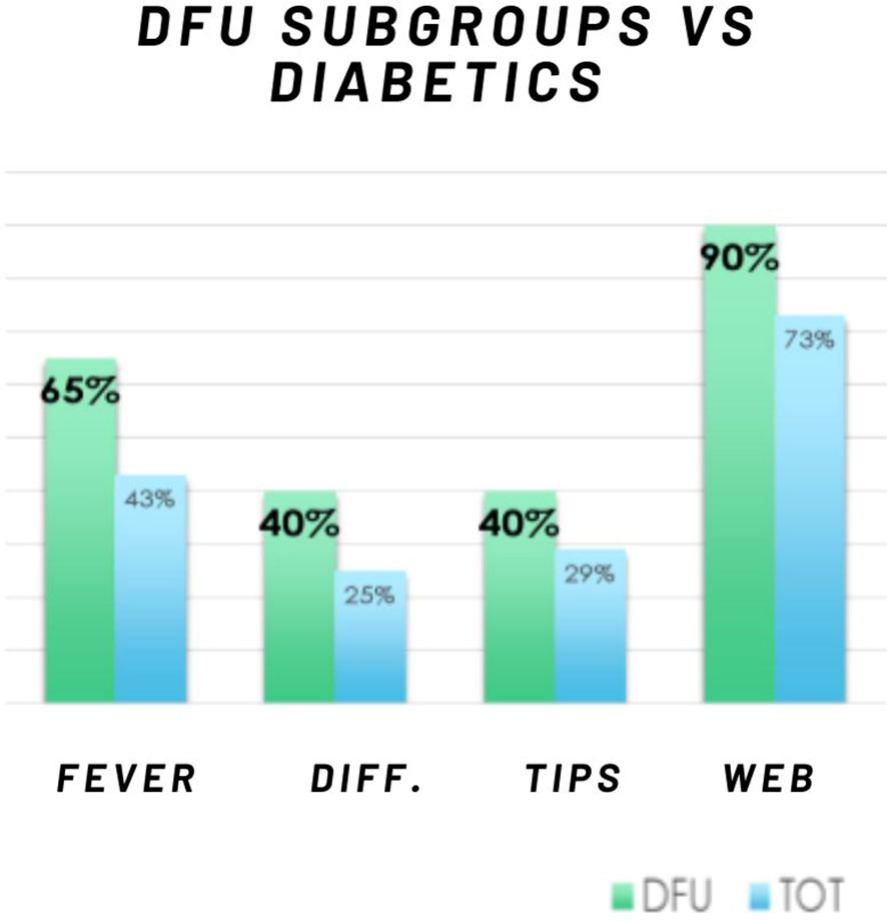
Comparison of risk awareness and information management: DFU subgroup vs. total diabetic patient population. DFU: ulcers, TOT: total, Fever: fever of tips, Diff.: management difficulty, Tips: remembering the rules, Web: web research.

In question 6, which assesses the timing and delivery of risk information for diabetic foot syndrome (**Figure 9**), it was found that 27% of participants received crucial information about their condition only after it had progressed to an advanced stage, specifically after the development of an ulcer. This alarming data highlights a significant gap in preventive care by health professionals, prompting an analysis of this subgroup, henceforth referred to as the DFU subgroup (the 27% who were first educated about the complications of diabetic foot after developing an ulcer).

The DFU subgroup shows that 65% of individuals in the DFU subgroup reported being troubled by the information provided by health professionals, compared to 43% of the total sample; 40% of the DFU subgroup (compared to 25% in the total sample) struggled with managing their foot care, indicating a gap in effective guidance; 40% of participants in the DFU subgroup (compared to 29% in the total sample) found it difficult to remember the instructions given by health professionals. A significant 90% of the DFU subgroup had searched for online resources about diabetic foot care, compared to 73% of the total sample. Moreover, all those who reported either not receiving information or being dissatisfied with the information they had received turned to online resources. Of these individuals, 75% reported understanding the information they found, compared to 64% of the overall sample.

These findings suggest important distinctions between the DFU subgroup and the total sample:

- Fear: A significantly higher percentage of individuals in the DFU subgroup experienced fear, indicating that the way information was conveyed may have induced anxiety, likely due to their advanced condition.
- Difficulties in management: The DFU subgroup also demonstrated greater difficulty in managing their feet. This may be attributed to ineffective communication of risk or an increased perception of challenges due to the advanced stage of their condition.
- Difficulty in remembering rules: The DFU subgroup showed a higher difficulty in remembering the guidance provided for managing diabetic foot care. This may suggest that post-ulceration information is perceived as complex or that these patients have mnemonic difficulties.
- Online search: The increased frequency of online searches among the DFU subgroup, as compared to the overall sample, indicates that patients who have already experienced complications are more likely to seek information and support online.

### 5.9 Interpretation of results and practical implications for health professionals

A simple survey to health professionals reveals a plethora of critical issues that undermine educational therapy and effective communication with the person being cared for.

A significant correlation exists between a diabetic’s understanding of their disease and their social class or level of education, as these factors often influence their ability to comprehend medical information.

Among the key barriers to effective communication is the use of technical jargon by healthcare professionals. Scientific and medical terms that are not readily understood by patients not only hinder comprehension but may also create a psychological barrier in the patient-provider relationship, impeding the development of trust and connection (18).

Zani, Selleri, and David (19) emphasize that patients are more likely to adhere to their therapeutic regimen if the information they receive is clear and unambiguous, which increases their satisfaction. Satisfaction is influenced by two behavioral models: the affective model (Zani and Cicognani) suggests that health professionals who demonstrate empathy and affective behaviors foster better patient engagement, while the cognitive model (Ley, 1989) posits that patient satisfaction is directly linked to the clarity and simplicity of the information conveyed.

To establish a strong connection with diabetic patients, proxemics—the physical distance between the patient and healthcare provider—plays a critical role. Psychological distance, influenced by physical proximity and body language, can significantly affect communication. Gestures and a welcoming attitude are crucial for building rapport. Additionally, the tone used should strike a balance between firmness and compassion, especially when discussing the severity of potential complications. Information should focus on practical strategies for preventing disease progression, avoiding fear-based communication that might exacerbate anxiety, depression, or self-sabotage (20).

The self-determination of the diabetic plays a critical role in their educational pathway. They must demonstrate a desire to improve their own health. Understanding the patient’s expectations from the relationship with health professionals helps clarify what they hope to gain from the interaction.

Patient education on daily podiatric care is fundamental, requiring clear, detailed instructions (21), along with printed and multimedia resources that can be referenced at home to aid retention.

The questionnaire also highlighted an important issue regarding the difference between public and private outpatient appointment scheduling. Public systems are often constrained by strict timelines, while private podiatric practices offer greater flexibility. These practices, by nature, provide a more comfortable environment for patients, thanks to the use of a podiatry chair, which promotes relaxation. This relaxed setting fosters effective educational therapy during both podiatric evaluations and treatments, increasing the time available for communication and education. Studies have demonstrated that learning is more effective when sessions are individual and consistent over time, allowing information to be broken down into manageable segments at each appointment (22).

Another key aspect of care is the use of therapeutic footwear and offloading devices (IWGDF 2023), which are critical for prevention but often rejected by patients due to discomfort. Encouraging the use of these medical devices is vital to protecting against complications associated with diabetic foot syndrome. Health professionals must emphasize the protective benefits of therapeutic footwear and offloading devices, as this knowledge is a cornerstone of effective patient education.

In addition, sensitizing family members, particularly caregivers, is essential. Caregivers, often responsible for daily patient support, must be properly trained in their role. This training ensures they can provide the necessary comfort, assistance, and cooperation with healthcare providers. Part of the patient interview should address how caregivers can effectively fulfill this crucial role (22, 23).

Communication barriers are not only present between patients and healthcare professionals but also within the multidisciplinary diabetic foot care team. Disparities in perspectives among team members—such as endocrinologists, internists, and diabetic foot specialists—can hinder collaboration. These professionals bring different skills and expertise, which must align to ensure comprehensive care for the patient. Creating effective interdisciplinary communication is essential for optimal patient outcomes.

It is crucial to dismantle these communication barriers within the healthcare team. Diabetic foot care is an urgent health issue, requiring coordinated efforts to provide high-quality care and establish a safe, supportive healthcare environment where patients feel protected, understood, and confident in their cooperation (24, 25).

Patient compliance is directly influenced by the healthcare professional’s communication and interpersonal skills, as well as their ability to establish a functional care network. Each team member plays a vital role in the network, contributing to the overall health system such as gears in a well-functioning machine (26, 27).

### 5.10 The role of ICT and barriers to implementation

Digital tools, such as teleconsultations and educational platforms, show considerable potential for improving communication in diabetic foot management. However, several barriers must be considered when integrating ICT into routine clinical practice.

One of the primary challenges is cost, as healthcare facilities and professionals may require financial resources to adopt new technologies, purchase digital devices, and ensure software maintenance. Additionally, disparities in digital literacy between healthcare professionals and patients pose a significant obstacle. While younger professionals and more technologically adept patients may easily adopt digital solutions, elderly individuals or those with limited technological skills may struggle to use these platforms effectively.

Another major challenge is institutional resistance to change. Healthcare organizations are often hesitant to adopt new digital tools due to concerns about workflow disruptions, potential technical issues, and the need for staff training. Furthermore, data security and regulatory compliance, such as adherence to GDPR and HIPAA regulations, add further complexity to the implementation of ICT in healthcare settings.

Recent studies Obilor et al., 2021 (10) have highlighted that ICT tools, including mobile applications, remote monitoring, and digital education programs, can significantly improve the prevention of diabetic foot ulcers, enhancing patient adherence and reducing the risk of complications.

### 5.11 Psychological and social factors in diabetic foot self-management

A study by Matricciani 2015 (21) found that diabetic foot self-management is often neglected by elderly individuals and is only adopted after complications arise. Their analysis identified key barriers, including physical ability, perceived importance, patient knowledge, availability of education, social integration, risk level, and communication with healthcare professionals. These findings reinforce the need for targeted therapeutic education programs that engage patients before severe complications develop.

Additionally, the World Health Organization (20) report on continuous training for healthcare professionals underscores the importance of structured therapeutic education to improve chronic disease prevention (Therapeutic Patient Education: Continuing Education Programmes for Health Care Providers in the Field of Prevention of Chronic Diseases).

## 6 Patient perspectives on information accessibility and gaps in professional training

### 6.1 Reliability of online information and its impact on doctor-patient communication

The literature review highlights that diabetic patients often seek online resources to fill the informational gaps left by communication with healthcare professionals. However, the quality of available information is highly variable, posing a significant risk of misinformation. According to Matricciani (21), poor health literacy and a lack of clear and accessible content contribute to the spread of incorrect self-management practices for diabetic foot care.

This issue is also reflected in the study by Curtis et al., 2013 (5), which found that communication training for healthcare professionals does not automatically translate into an improved patient experience. If patients perceive communication with doctors as insufficient, they may be more inclined to seek information elsewhere, increasing their exposure to unreliable sources.

A study by Obilor et al., 2021 (10) examined the use of digital technologies for diabetic foot ulcer prevention, revealing that mobile applications and online platforms can be effective tools for improving treatment adherence. However, the lack of standardization in the content available online limits their effectiveness.

To further justify the implementation of the Diabetic Foot Talk-Time Connect framework, it would be beneficial to analyze the digital sources currently used by patients. This would help determine whether reliable tools already exist or if there is a need to develop more accessible and trustworthy resources, integrated with direct support from healthcare professionals.

### 6.2 Availability of standardized programs for diabetic foot communication in medical education

The literature review indicates that there are currently no standardized programs dedicated specifically to communication in diabetic foot management within medical and nursing curricula. Communication training in clinical education is generally incorporated into medical courses in the form of general training on doctor-patient relationships and delivering bad news, but it rarely addresses the specific challenges of managing chronic conditions such as diabetes.

The study by Curtis et al. (5) demonstrated that communication training through practical simulations can improve healthcare professionals’ skills, yet it does not necessarily lead to positive patient outcomes. This suggests that a communication training program should be accompanied by practical tools that facilitate the application of acquired skills.

The World Health Organization (20) report on continuous training for healthcare professionals underscores the need for targeted educational programs to improve chronic disease prevention. This supports the idea that the Diabetic Foot Talk-Time Connect framework could not only enhance communication between patients and healthcare professionals but also serve as a training tool for practitioners.

### 6.3 Framework

The management of diabetic foot syndrome (DFS) poses unique challenges, not only in medical intervention but also in fostering effective communication between healthcare professionals, patients, and caregivers. Insights gathered from the preceding analysis highlight significant gaps in educational communication, preventive care, and multidisciplinary collaboration. These issues underscore the necessity of a structured framework to improve the quality and clarity of interactions across all facets of diabetic foot care.

This framework emerges as a response to the recurring barriers identified throughout the study. It is built on evidence-based practices and real-world scenarios, providing healthcare professionals with practical tools and strategies to enhance communication and patient education. By addressing the diverse needs of diabetic patients, caregivers, and interdisciplinary teams, the framework seeks to create a cohesive, patient-centered approach to diabetic foot management.

The upcoming chapter details a structured framework that aims to:

- **Streamline Communication**: Offer practical recommendations for clear and effective dialogue with patients and caregivers.
- **Enhance Preventive Education**: Equip individuals with the knowledge and resources needed to proactively manage their condition.
- **Foster Multidisciplinary Collaboration**: Provide strategies to unify the efforts of healthcare teams, ensuring seamless coordination and comprehensive care.
- **Empower Patients**: Encourage self-management and adaptive problem-solving through accessible and actionable guidance.

Rooted in empathy and simplicity, the framework aspires to redefine communication standards in diabetic foot care, bridging gaps that currently hinder effective management. By integrating this model into practice, healthcare professionals can contribute to the prevention of complications, the improvement of patient outcomes, and the advancement of diabetic foot care on a systemic level.

**Figure 10:**
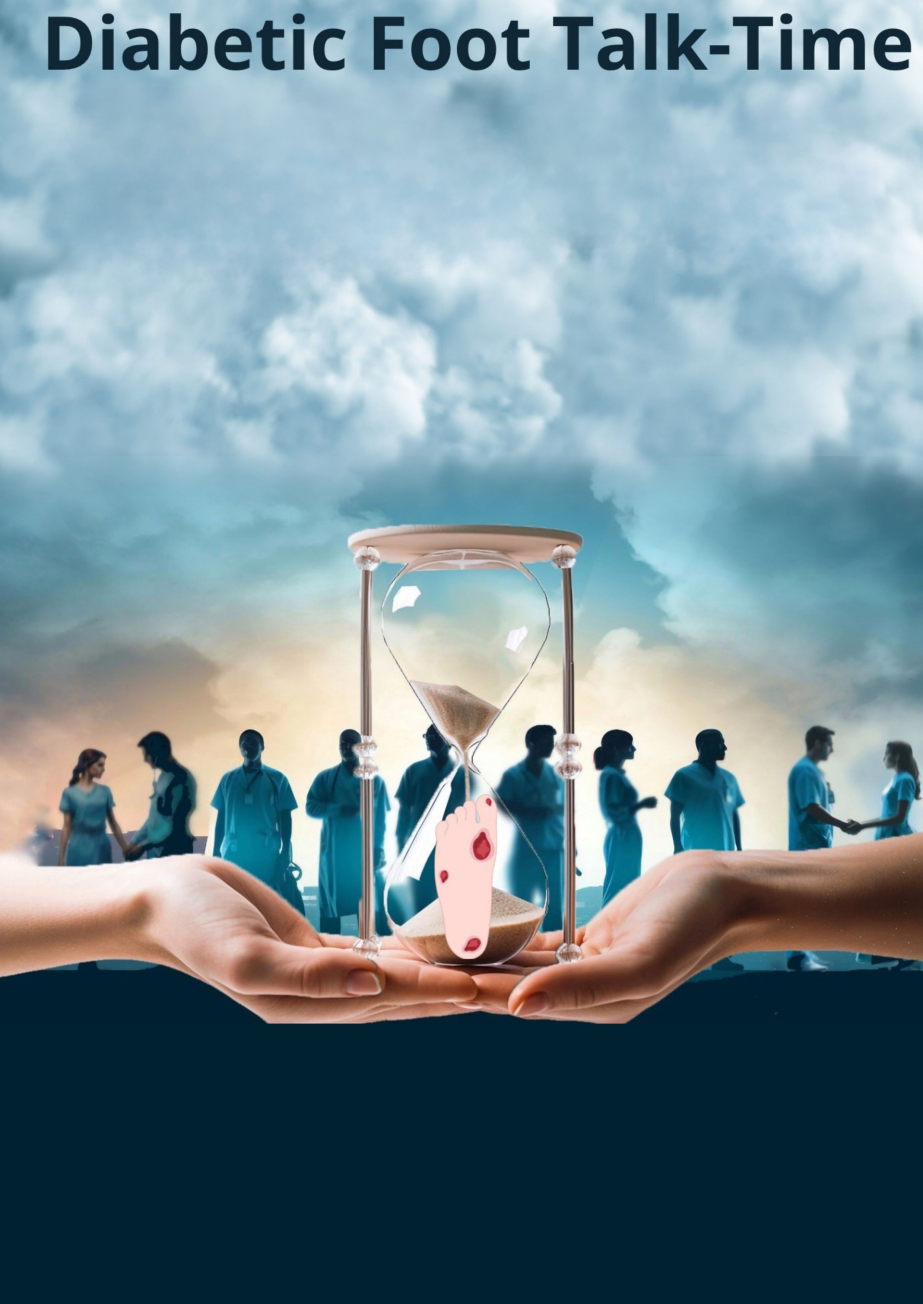
Representative image of the framework.

The image is intended to be representative of the theme; in the background, a group of healthcare professionals is seen interacting with each other and empathically with patients, conveying the idea of effective, interprofessional communication (**Figure 10**).

The two hands in the foreground, holding an hourglass, symbolize Time in the widely used medical analogy, “Time is Tissue,” particularly relevant in the context of diabetic foot care. This analogy underscores the critical importance of immediate intervention. Emerging from the continuous flow of sand is a foot with multiple lesions, serving as a visual warning of the risks associated with the condition.

#### 6.3.1 Framework structure

The framework serves as a conceptual model, organized into chapters for simplicity. Each chapter addresses specific aspects of effective communication, guiding readers through essential topics, including:

- Practical recommendations for establishing effective communication with diabetic foot patients during healthcare interviews, covering the healthcare setting, proxemics, and verbal communication.
- Key questions to assess a patient’s understanding of their condition and emotional response.
- Clear, concise explanations about the risks and complications of diabetic foot syndrome.
- A daily podiatric routine, offering easy-to-memorize recommendations for caregivers, starting with the initial encounter and continuing with updates from the IWGDF 2023 during subsequent visits.
- Information to build a trusting, effective relationship with caregivers.
- Strategies to enhance communication with other healthcare professionals.

Each chapter includes real-life scenarios and practical exercises, designed to improve communication skills in complex situations. These scenarios emphasize empowering diabetic patients by guiding them toward problem-solving and adaptive behaviors in response to unexpected challenges.

### 6.4 How to approach the health interview

This section focuses on the health interview—pragmatically, the approach to dialogue and relationship-building with diabetic patients.

Literature suggests that certain foundational principles must be followed to achieve the desired communication outcomes, although these may vary depending on context and specific needs.

First, a clear introduction is vital. In any relationship, this initial step precedes the interview, setting the stage for a successful therapeutic alliance. The patient must understand who they are speaking to, the competencies of the professional, and their role within the interdisciplinary team.

Next is the organization of the therapeutic and communicative setting, which plays an active role in communication. This environment must be conducive to the relationship: it should not be distracting, excessively distant, or noisy, as these factors can hinder effective dialogue. If the setting cannot be controlled, healthcare personnel should strive to minimize physical distance and ensure the patient feels at ease, compensating for any environmental shortcomings.

During the first interaction with the patient, it is important to maximize empathic skills, making an effort to understand their perspective. A smile helps establish initial nonverbal contact, which should be followed by creating an appropriate “proxemic space”—the right interpersonal distance. This ensures the patient feels neither too distant nor overwhelmed, signaling warmth and a sense of commonality.

During the dialogue, it is crucial to use an appropriate language register and avoid technical-scientific terminology. Professionals have identified educational level and socio-cultural differences as major barriers to effective communication. To enhance understanding, it is essential to simplify the message, using terms that are easily comprehended. This is supported by both the literature and professionals’ responses to survey question 11, which highlighted these barriers. Effective communication also requires avoiding distractions caused by misunderstandings, using a firm yet reassuring tone to emphasize the importance and truthfulness of the message. The tone should not instill undue fear, nor should it be too friendly, as this could lead to confusion or cause the diabetic patient to underestimate the message.

These principles structure communication, ensuring its effectiveness by simplifying the message in a way that is easily remembered. The primary goal is to establish a strong initial connection with the patient.

Following the health assessment, a cognitive and circumstantial evaluation is necessary to understand the patient’s thoughts and emotions related to their disease and foot care. This includes interpreting their fears and actions to better understand them and strengthen the helping relationship.

In many cases, diabetics are not properly trained in foot care management, whether or not they are considered at risk for complications. It is possible that no one has ever explained the risks associated with diabetic foot or the necessary preventive measures. Alternatively, they may have received this information only after experiencing their first complication. This is reflected in survey data, where 27% of diabetics reported inadequate education on foot care. Additionally, 73% of respondents searched for information online, and 90% of those who were educated about diabetic foot care received this information only after an ulcer had developed.

“There is substantial consensus in the literature that communication during clinical consultations serves three primary functions:

1. Collect information;
2. Provide information;
3. Create a relationship.

Typically, information gathering takes place at the beginning of the consultation, with information delivery following. Relationship-building, however, is continuous and evolves throughout the consultation. A key distinction is that while the skills required for gathering and providing information are well-defined and teachable, techniques for relationship-building are more complex. Many experts agree that a strong, empathetic relationship is based on intangible factors, such as mutual respect and genuine concern for the patient’s struggles and challenges.

II Module “Physician-Patient and Health Professional Communication” - Office III - Communication and Professional Performance: Methods and Tools, Ministry of Health - Health Programming Direction General, page 18.

The responses to the eleventh item of the questionnaire for health professionals highlight key obstacles to effective communication with diabetic patients, emphasizing two major factors: the underestimation and dispersion of the risks associated with diabetes, and the denial of the clinical condition itself. This reflects a widespread misconception among diabetics, many of whom believe that the disease only exists when complications arise. Specifically, they may only acknowledge their condition when severe health impairments occur, often triggered by visible complications such as amputations or the onset of diabetic foot syndrome. Prior to this point, oral medications might be seen as less serious, creating the impression that diabetes, especially in its early stages, is not a serious condition. This erroneous belief extends to diabetic foot syndrome, where many patients fail to make the critical connection between their diabetes and the risk of foot complications until ulcers or more significant lesions appear. Furthermore, the emotional and psychological challenges of accepting a chronic condition like diabetes are substantial. For many patients, acknowledging their condition requires significant emotional adjustment. Denial, apathy, and resistance to change are common responses, particularly because of the tangible difficulties involved in managing the disease. Many perceive diabetes as a limitation to their independence and lifestyle, which compounds the emotional burden of accepting the necessary lifestyle changes. This psychological barrier is often compounded by a lack of awareness or understanding of the broader implications of the disease.

The following questions are designed to ask the patient.

**Questions**

- DO YOU KNOW YOUR CONDITION “DIABETES”? WHAT DO YOU THINK ABOUT IT?
- HAVE YOU CHANGED YOUR DAILY LIFE?
- DO YOU HAVE A FAMILY MEMBER TO HELP YOU?
- HAVE YOU RECEIVED INFORMATION ABOUT THE RELATIONSHIP BETWEEN DIABETES AND DIABETIC FOOT?
- WHAT DO YOU THINK DIABETIC FOOT MEANS?
- DO YOU KNOW THE RISKS OF DIABETIC FOOT?
- DO YOU KNOW WHAT DIABETIC NEUROPATHY MEANS?
- DO YOU KNOW THE MEANING OF LOWER LIMB ARTERIOPATHY?
- DO YOU KNOW WHAT DIABETIC ULCERS ARE?

The answers, to such questions, guide the healthcare professional in understanding the patient’s caregiver, personal, and social identity. The responses provide critical insights into the patient’s relationship with the disease, their emotional state, their knowledge of the syndrome, and their acceptance of it. These answers become essential cognitive tools for effective care when interpreted correctly, as they reveal the degree of distress, beliefs, and emotional reactions.

Active listening is vital at this stage; healthcare personnel should demonstrate complete engagement with the patient’s views, both explicit and implicit, while suspending all judgment. The patient must feel genuinely understood and not judged.

This approach lays the foundation for determining the appropriate care pathway and for initiating a process of redefinition of the patient’s relationship with diabetes.

The vademecum accompanying each question will include explanations on the disease and its complications. These explanations are crafted to follow the principles of simplicity, clarity, and ease of recall. It is crucial, especially during the first consultation, to avoid projecting exaggerated future risks. As noted in responses to question 11 of the health professionals’ questionnaire, fear can impede effective communication by inducing distress and anxiety, ultimately damaging the patient-care provider relationship. It is therefore essential to present potential complications alongside reassuring information to mitigate these concerns.

### 6.5 Explanation

#### 6.5.1 Relationship between diabetes and the diabetic foot

“Diabetes is a condition that affects the body’s ability to regulate blood sugar. This can impact various parts of the body, including the feet. The term ‘diabetic foot’ refers to the foot problems that people with diabetes may experience.”

#### 6.5.2 Nerve damage (diabetic neuropathy)

“Diabetes can damage the nerves in the legs and feet. Diabetic neuropathy may cause loss of sensation in the feet, leading to tingling or numbness. This can make it difficult to feel wounds or injuries, increasing the risk of unnoticed foot damage. It is crucial to regularly inspect your feet for any signs of injury.”

#### 6.5.3 Decreased blood flow (peripheral arteriopathy)

“Diabetes can impair circulation to your legs and feet. Poor blood flow makes it harder for wounds to heal and increases the risk of infections. As a result, your feet may become cold, painful, or swollen.”

#### 6.5.4 Ulcers and infections

“Because diabetes can affect both nerve function and blood flow, it may lead to foot ulcers—open, painful wounds that are susceptible to infection. These infections can spread quickly and lead to serious complications. Additionally, diabetes weakens the immune system, making it more difficult for your body to fight off infections, which can be life-threatening.”

#### 6.5.5 Amputations

“In severe cases, if complications aren’t treated early, part of the foot or leg may need to be amputated. Preventing this outcome is crucial, which is why it’s essential to prioritize proper foot care.”

#### 6.5.6 Self-management and prevention

“The good news is that you can take steps to prevent these complications. Inspect your feet daily, keep them clean, and moisturize them regularly. Always wear comfortable, well-fitting shoes.”

#### 6.5.7 Physician involvement

“You don’t have to manage this alone. If you notice any changes in your feet, consult with your doctor or a podiatrist. They can guide you in managing any issues that arise.”

Using clear, simple language and concrete examples significantly improve understanding. can

Visual aids, such as images illustrating the consequences of poor foot care (without being too graphic), can make the risks more tangible and motivate patients to follow their care regimen.

It is important to evaluate whether the message has been effectively communicated. After educating caregivers about the risks of diabetic foot syndrome, asking follow-up questions can help assess their understanding.

### 6.6 Evaluation of effectiveness

- WHAT DO YOU THINK OF THIS INFORMATION?
- DO THEY SCARE YOU? WHAT ARE YOU MOST CONCERNED ABOUT?
- DO YOU HAVE ANY QUESTIONS?
- HOW CAN I HELP YOU?

This feedback allows healthcare professionals to adjust their strategies to ensure clarity and set realistic, measurable goals for both patients and caregivers. Sharing success stories from other diabetics who have successfully managed their condition can inspire hope and encourage patients to adhere to their treatment plans.

After pathology acceptance and identity confirmation, a key objective for healthcare personnel is the implementation of diabetic-podiatric education. This is crucial for initiating self-care by providing essential information on daily foot hygiene, monitoring, and lifestyle modifications. Multiple RCTs in the literature have demonstrated the effectiveness of these interventions in improving patient outcomes.

**Questions**

- IS IT DIFFICULT TO CONTROL YOUR FOOT?
- HAVE YOU EVER HAD DISCOMFORT, DISCOMFORT OR PAIN IN YOUR FEET?
- DO YOU KNOW THE RULES FOR FOOT CARE?

**PodoDaily routine: Information on positive behaviors to promote in diabetic patients**

Data from a survey of diabetics revealed that 58% recall 1 to 5 key rules for foot care, 24% remember 1 to 3, and only 18% can recall 6 to 10. This variation highlights the challenge in helping patients absorb and retain important care guidelines. Most patients tend to remember only the most significant or easier-to-follow rules. To address this, a simple, structured daily foot care routine has been designed, based on the SID Decalogue (2019) and the Epitech group’s diabetic foot rules (adapted from Brodsky, 1993). This routine will be introduced and reinforced during follow-up visits to improve patient education and adherence to self-care practices.

### 6.7 PodoDaily introductory routine

In line with the 2023 guidelines, the following simple recommendations are provided:

#### 6.7.1 Do not walk barefoot

- Recommendation: Avoid walking barefoot, even indoors, to protect your feet from possible injury.

#### 6.7.2 Foot inspection

- Recommendation: Inspect your feet daily for cuts, blisters, calluses, or redness. Use a mirror, if needed, to check the bottoms of your feet.

#### 6.7.3 Thorough foot washing and hydration

- Recommendation: Wash your feet daily with warm (not hot) water and mild soap. Dry thoroughly, especially between the toes. Apply moisturizer to the feet, avoiding the spaces between the toes, to prevent dryness and cracking.

#### 6.7.4 Nail hygiene

- Recommendation: Trim toenails straight across and avoid cutting them too short. Use a nail file instead of scissors to prevent cuts or infection.

#### 6.7.5 Appropriate footwear

- Recommendation: Always wear footwear prescribed by a specialist. Ensure shoes are comfortable, well-fitting, and free from tightness or inner seams that could cause harm.

#### 6.7.6 Regular monitoring and timely reporting

- Recommendation: Keep regular appointments with your podiatrist and physician for checkups and preventive care. Promptly report any foot injuries, cuts, or changes to a healthcare professional.

### 6.8 Feedback on routine

WHAT DO YOU THINK YOU CAN’T DO?

If the person receiving assistance experiences difficulty with these activities, obstacles can be overcome by involving a caregiver. More often, diabetes patients have various limitations and difficulties in carrying out some important rules due to age or pathology, which is why it becomes setal the intervention of the caregiver in their daily life, who will attend the interviews to become educated as the assisted person with respect to all recommendations and advice to be followed during the course of care implementing its adherence.

It is also important to ensure availability and effective response to the caregiver’s questions, providing ongoing empathic support.

Finally, it’s essential to recognize and celebrate any improvement in foot care, no matter how small. Positive reinforcement through praise enhances motivation and increases compliance.

### 6.9 Alarm bells: signs and symptoms for early recognition

Early recognition of diabetic foot complications and associated risks is crucial to effective care. Health professionals must educate individuals with diabetes about the warning signs that signal the need for early diagnosis and intervention. To facilitate this, key signs and symptoms for recognizing complications are presented in a straightforward, easy-to-understand formula:

1. Tingling and Numbness: Diabetic neuropathy, a common complication, can cause tingling, numbness, or a “pins and needles” sensation in the feet and legs. This may be accompanied by a reduced ability to sense temperature changes or simultaneous sensations of hot and cold. These symptoms are often early indicators of nerve damage.
  - Sensation of walking on cotton
  - Sensation of needles and pins
  - Feeling of fires and refrigerators underfoot
2. Dry and Cracked Skin: Dry, cracked skin is a common sign of skin dehydration, especially in diabetic patients, and can increase vulnerability to injury and ulcers. Adequate skin hydration is essential for prevention.
3. Callosities or Calluses: Thickened skin or calluses on the feet, often caused by abnormal pressure, friction, or ill-fitting footwear, can increase the risk of developing ulcers. Left untreated, these areas may become more susceptible to injury and infection.
4. Blisters or Wounds: Non-healing blisters or wounds may be signs of poor circulation or neuropathy. Such conditions are critical warning signs that should not be ignored.
5. Thickened, Curved or Ingrown Nails: Thickened or curved nails, as well as ingrown nails, can lead to injury or infection, requiring immediate attention to prevent complications.
6. Changes in Coloration: Alterations in skin color, such as paleness or redness, may be signs of circulation issues or excessive pressure from footwear, requiring close monitoring.
7. Calf Pain: Pain in the calves, particularly at night or after short walks, may indicate insufficient blood flow to the legs, a condition known as peripheral artery disease (PAD).
8. Swelling: Swelling of the feet, ankles, or legs can be a sign of fluid retention, poor circulation, or inflammation, and may signal underlying health issues.
9. Changes in Skin Temperature: An abnormal change in foot temperature, such as excessive warmth or coolness compared to the rest of the body, could indicate circulatory or inflammatory problems.
10. Changes in Foot Shape: Diabetic foot deformities, such as hammer toes, hallux valgus, metatarsal protrusion, or a hollow foot, can alter pressure distribution and increase the risk of ulcer formation.
11. Loss of hair: Reduced hair growth on the feet or legs, particularly in men, may be a sign of poor circulation and should be evaluated as part of regular foot care.
12. Muscle Weakness: Neuropathy can lead to muscle weakness in the feet and legs, which may manifest as difficulty walking or maintaining balance. This requires attention to prevent falls or further complications.
13. These symptoms can vary between individuals. If a diabetic patient experiences one or more of these signs, immediate consultation with a healthcare professional, such as a podiatrist or diabetologist, is crucial for evaluation and treatment. Diabetic patients should be encouraged to perform regular self-examinations of their feet and report any abnormalities or concerns promptly. Prevention, continuous monitoring, and early intervention are key to preventing serious complications.

### 6.10 Subsequent meetings

The literature and the IWGDF 2023 guidelines emphasize the importance of structured, ongoing therapeutic education for caregivers. As reported by questionnaires, 26.8% of professionals discuss the risks of diabetic foot during follow-up visits or after risk stratification. Additionally, 19.2% of diabetic patients report receiving information on the significance of diabetic foot only in later visits. These findings suggest that, while initial education may be insufficient, follow-up visits present an opportunity to improve patient understanding and prevent complications through continued education.

#### 6.10.1 Recommendations for subsequent meetings

Moderate exercise:

- Recommendation: Regular physical activity, such as walking, is essential for maintaining overall health and circulation, particularly for individuals with diabetes.
7. Control of ulcers:
- Recommendation: Wounds or ulcers should be monitored closely. If any changes occur, immediate consultation with a healthcare provider is recommended for timely intervention.
Offload:
- Recommendation: Always wear the acute-phase footwear (discharge shoe) prescribed by healthcare professionals when a plantar, dorsal, or digital ulcer is present.

#### 6.10.2 Additional recommendations for at-risk foot

If a diabetic patient is classified as risk class 1 or 2, it is essential to educate them on preventative measures to avoid the onset of ulcerative lesions or other complications.

Temperature:

- Recommendation: If signs of inflammation—such as swelling, heat, or reduced mobility—are present, check the temperature of your feet once daily. If the temperature of one foot differs by 2°C from the other for 2 consecutive days:
- walk less
- call your referring diabetologist/endocrinologist or a podiatrist

Consider educating at-risk diabetic individuals on monitoring these temperature changes.

Foot-ankle exercises:

-Recommendations: Follow a foot-ankle exercise program for 8–12 weeks under the guidance of a trained professional.

1000 Steps:

-Recommendations: Limit walking to no more than 1,000 steps per day.

If longer walks are necessary for work or leisure, ensure you wear the appropriate footwear and monitor your feet regularly.

(Today, smartphones and smartwatches feature step-counting apps, or you can advise caregivers to use a simple pedometer.

Continuing education:

-Recommendation: Continuously educate yourself on diabetic foot care. Adhere to the guidelines provided by your medical team.

### 6.11 Content web page dedicated to the assisted person

Clear, concise explanations of diabetic foot syndrome’s risks and complications, including often overlooked or misdiagnosed signs.

Simple, easy-to-remember daily podiatric practices with illustrative images for effective self-care.

Early detection signs and symptoms of diabetic foot syndrome complications.

### 6.12 Comparison with existing digital tools and innovation of the proposed framework

Recent advancements in mobile health (mHealth) applications have contributed significantly to diabetic education, particularly in the domain of diabetic foot syndrome (DFS) prevention and monitoring. However, a closer examination reveals that existing platforms often address only isolated components of diabetic foot care.

Several apps, such as Diabetic Foot Smart (28) and MyFootCare (29), focus predominantly on monitoring ulcer progression or identifying risks for ulceration. For instance, the MyFootCare app facilitates the self-monitoring of diabetic foot ulcers by enabling users to photograph and track wound healing through visual analytics. While users perceive it as valuable, engagement issues often arise due to usability barriers, technological literacy challenges, and emotional distress linked to a lack of perceived healing (29).

While these applications provide meaningful innovations, they lack a structured, holistic communication framework that bridges patient education, caregiver support, and interdisciplinary healthcare collaboration. They tend to focus narrowly on ulcer size tracking, without integrating comprehensive strategies for behavioral reinforcement, emotional empowerment, caregiver training, and interprofessional dialogue.

The “Diabetic Foot Talk-Time Connect” framework advances beyond these existing models by:

- Offering multimodal educational content (visual, behavioral, verbal) accessible online and offline, ensuring inclusivity even for those with limited internet access.
- Embedding caregiver education systematically into the communication pathway, recognizing the central role caregivers play in patient adherence.
- Structuring interdisciplinary communication to align strategies among diabetologists, podiatrists, nurses, and other specialists.
- Addressing emotional and motivational dimensions of care, moving beyond clinical surveillance to patient empowerment.
- Being founded on real-world survey data capturing communication challenges faced by both patients and professionals, ensuring that the framework is deeply rooted in lived experiences.

This integrated and dynamic approach represents a transition from passive monitoring to an active, empowering care model that fosters shared responsibility among all stakeholders. In contrast to the primarily monitoring-focused tools currently available, “Talk-Time Connect” positions communication as a therapeutic tool itself, crucial for successful DFS prevention and management.

In summary, by integrating behavioral science, communication theory, practical routines, and the use of technology into one cohesive model, the framework fills critical gaps unaddressed by existing applications. It offers an innovative paradigm shift: from “monitoring disease” to actively managing communication for prevention, empowerment, and healing.

### 6.13 Limitations of the study and future directions

This framework introduces a pioneering approach to effective communication in diabetic foot management. A significant challenge in applying this model stems from technological accessibility. Not all healthcare professionals or patients have access to internet-enabled devices or smartphones, which could hinder the effectiveness and reach of online resources such as QR codes. While the framework strongly emphasizes digital tools to enhance education and communication, we acknowledge that not all patients and healthcare professionals have consistent access to the internet or possess sufficient digital literacy. To address this, the framework could be complemented by offline resources such as printable educational brochures, visual aids, and structured interview checklists for in-person consultations, ensuring inclusivity and broader reach.

Future studies should broaden the sample size of healthcare professionals and diabetic patients to enhance the representativeness and generalizability of the results. Clinical implementation and evaluation of the framework’s effectiveness in improving communication and patient outcomes are essential. Additionally, research should assess the impact of QR codes on the interaction between healthcare professionals and patients, monitoring changes in clinical practice and patient understanding. Feedback channels should be created to gather insights from users accessing content through these digital platforms.

This project serves as a foundation for improving diabetic foot care, emphasizing informed and collaborative management. The framework aims to foster better communication between diabetic patients and healthcare teams, with a shared focus on prevention and effective management.

In an increasingly digital world, online resources are a critical step forward in promoting education, prevention, and communication to reduce complications related to Diabetic Foot Syndrome. The long-term goal is the creation of an innovative smartphone application that will revolutionize diabetic foot management. This comprehensive tool will support prevention, monitoring, and management, benefiting both caregivers and healthcare professionals and establishing a coordinated network of care.

This approach promises a more effective future for managing this complex syndrome. Although primarily focused on diabetic foot care, this framework can be adapted for other chronic conditions, including wound management and peripheral artery disease. A roadmap for scaling and integrating the framework into broader healthcare ecosystems is included.

A pilot study is planned in collaboration with diabetic foot clinics across Italy, followed by institutional partnerships for funding and potential integration into national healthcare systems. Performance metrics, including patient adherence rates and clinical outcomes, will guide iterative framework improvements. Funding opportunities and potential collaborations with digital health firms are being explored to support long-term implementation.

The framework will comply with GDPR and HIPAA regulations to ensure the security of patient data. Furthermore, informed consent protocols and ethical considerations regarding AI-assisted healthcare decision-making will be incorporated.

Additionally, the reliance on self-reported data regarding patient adherence and understanding introduces potential biases, including recall bias and social desirability bias. This limitation may affect the accuracy of reported behaviors and perceptions. Future studies should consider incorporating observational data or objective adherence metrics where possible.

## Data Availability

The original contributions presented in the study are included in the article/supplementary material. Further inquiries can be directed to the corresponding author.
