# Supplementary material for: Diabetic Foot Talk-Time: framework for effective communication in diabetic foot management
Source: Front Clin Diabetes Healthc. 2025 Jun 23;6:1590570. doi: 10.3389/fcdhc.2025.1590570 (PMC12232915; doi:10.3389/fcdhc.2025.1590570)
Supplement: Supplementary file 1 [file Table1.docx]

1. **Questionnaire dedicated to health professionals**

**Items:**

**Section 1 Information about the practitioner**

1. Profession

- Endocrinologist/Diabetologist
- Infectivologist
- Cardiologist
- Anesthesiologist
- General surgeon
- Orthopedic surgeon
- Plastic surgeon
- Vascular surgeon
- Interventional radiologist
- Physiatrist
- General practitioner
- Podiatrist
- Nurse
- Physiotherapist
- Orthopedic technician
- Psychologist
- More

1. Where do you practice your profession?

- Department of Endocrinology, Internal Medicine, Diabetology, Surgery, Orthopedics, Physiatrics
- Outpatient Clinic/Diabetic Foot Center
- Private Practice
- Outpatient Department of Primary Care Medicine
- more

1. Are you included in the Multidisciplinary Diabetic Foot Team?

**Section 2 Evaluation of current communication**

1. At what stage of the health interview do you explain the risks of diabetic foot?

- During the diagnosis of diabetes
- During subsequent visits
- After stratification of the at-risk foot
- After the first access for ulceration

1. Do you provide paper support materials to the patient?
2. What do you think is the most important information to provide to the diabetic with an at-risk foot?

- The complications he may face
- The daily routine of podiatric care
- Actions to avoid

1. Do you devote time to interviewing the "caregiver," the one who cares for the person assisted with diabetic foot on a daily basis?

**Section 3 Expectations**

1. Do you think the diabetic understands after the health interview the complications he or she might face if he or she does not follow the suggested advice?
2. Do you feel that your patients follow the advice given during visits?

**Section 4 Challenges and obstacles to communication**

1. How difficult do you think, on a scale of 0 to 10, where 0 is not at all difficult and 10 is very difficult, is communication with the person assisted with diabetic foot?
2. What do you think are the biggest obstacles to communication with the person being cared for?
3. What do you think the assisted person does not understand, to a greater extent, about their condition?
4. What about what they are told?

**Section 5 Strategies for improving communication**

1. How can we help him?
2. Do you think the assisted person could learn to better adhere to the rules on diabetic foot management if they could consult them online by connecting with their smartphone to a dedicated web page?

**Section 6 Challenges to interdisciplinary collaboration**

1. What do you think is a limitation in communication with other professionals in the Interdisciplinary Diabetic Foot Team?
2. What about those who are not part of the Team?

**Section 7 Suggestions and improvements**

1. What do you think could be improved in communication between professionals?
2. Do you think specific training courses are needed to improve communication with other professionals?
3. **Questionnaire dedicated to diabetics**

**Section 1 Risk awareness**

1. Do you know the risks of diabetic foot?
2. Do you know what diabetic neuropathy means?
3. Do you know the meaning of lower limb arteriopathy?
4. Do you know what diabetic foot ulcers are?

**Section 2 Communication and education**

1. Do you remember whether this information was given to you by the health staff during your visits?
2. When, for the first time, did they explain to you what diabetic foot is?

- At the diagnosis of diabetes
- During subsequent visits
- When he already had an ulcer
- Never

1. Did they explain how to manage your foot?

**Section 3 Feedback and satisfaction**

1. Did you think the information you received was complete? Did they satisfy you?
2. Did the information the health staff provided scare you?
3. Did they also give you paper support materials?
4. Did you Google for information?
5. Do you think you understood the information found?
6. Do you have difficulty in managing your foot?
7. Do you think it is difficult to follow the rules given to you by the health staff?
8. And remember them?
9. How many rules can you remember?

- 1 to 3
- 1 to 5
- 1 to 10

1. Were you able to put them into practice?
2. Do you find the suggested rules difficult to put into practice?

**Section 4 Suggestions for improvement**

1. Would it be easier to consult the rules with your smartphone (cell phone)?
2. Does anyone help you in daily life?
