# Supplementary material for: Diabetic Foot Talk-Time: framework for effective communication in diabetic foot management
Source: Front Clin Diabetes Healthc. 2025 Jun 23;6:1590570. doi: 10.3389/fcdhc.2025.1590570 (PMC12232915; doi:10.3389/fcdhc.2025.1590570)
Supplement: Supplementary file 2 [file Table2.docx]

**Complete Responses to the Qualitative Items to the Health Professionals' Questionnaire**

**11. What do you think are the main obstacles to communication with the patient?**

| **Responses** |
| --- |
| Fear. |
| Socio-cultural level. |
| Long-standing bad habits. |
| Social class. |
| The patient does not realize how insidious and dangerous their disease is. |
| Acceptance of the disease. |
| Diabetes is always underestimated by patients, and they do not consider the consequences. |
| Superficiality. |
| Underestimation of the dangers they might face. |
| Incorrect communication. |
| Lack of a caregiver. |
| Language barriers. |
| Not being understood. |
| Limited cultural education, tendency to underestimate the disease, limited cooperation among professionals, and limited financial resources. |
| Underestimation of the problem. |
| Lack of awareness of the risks until they experience severe complications like ulcers. |
| Communication issues. |
| Non-acceptance of the disease/refusal to acknowledge complications or undergo treatment. |
| Patients downplaying the seriousness of their condition. |
| Poor compliance. |
| Most diabetic patients do not accept the disease and consequently underestimate potential damage. |
| Age. |
| Patient disinterest. |
| The educational level of the patient. |
| Lack of knowledge about the disease. |
| Patients pretending not to see or hear the issues. |
| Limited time availability. |
| Resistance to treatment from diabetic patients, tired of an invasive disease. |
| The topic itself, such as discussing unseen risks like tight shoes or long foot baths. |
| Lack of attention to the problem. |
| Elderly patients left to fend for themselves without help, or those who do not comprehend the importance of diabetic foot care. |
| Lack of understanding of the real risks. |
| Indifference to the problem. |
| Non-acceptance of the condition, creating psychological barriers. |
| Poor compliance and lack of understanding. |
| Cultural beliefs, such as the use of inappropriate footwear. |
| Overreliance on the internet for information. |
| Ignorance about the subject. |
| Difficulty understanding the risks of the disease. |
| Lack of information and prevention. |
| Belief that diabetes is not a serious disease. |
| Anxiety. |
| Lack of patient awareness of the condition’s seriousness. |
| Poor comprehension of the complexity of their condition. |
| Cognitive, educational, social, and cultural factors. |
| Empathy barriers, such as feeling judged. |

**12. What do you think the diabetic foot patient does not understand most about their condition?**

| **Responses** |
| --- |
| The complexity of their condition. |
| Prevention. |
| The importance of proper footwear. |
| Further complications. |
| The severity of foot lesions. |
| The rules to follow. |
| The complications. |
| The importance of proper nutrition and footwear. |
| Risks associated with ulceration. |
| How prevention can stop rapid deterioration. |
| Lack of proper information. |
| Neuropathy: mechanisms and causes. |
| Risks from simple actions like improper toenail cutting. |
| The danger of ignoring their condition. |
| Why diabetes, as a “silent” disease, can cause significant harm. |
| The importance of glycemic control and appropriate footwear. |
| The irreversible nature of complications. |
| The seriousness of the condition’s progression. |
| Lack of pain leading to reduced attention. |
| The chronic nature of diabetes and why prevention matters before complications arise. |
| They underestimate the risks surrounding diabetes. |
| The risk of ulceration due to improper footwear. |
| The gravity of consequences linked to their condition. |
| Issues related to altered sensitivity. |
| The potential for severe damage if unmanaged. |
| Why lifestyle choices directly impact their quality of life. |
| The risks of poor compliance, including ulceration and amputation. |
| Patients with fewer financial resources or associated depression are often the least stable in their condition. |
| They often fail to accept the disease. |
| Psychological, social, and economic barriers to understanding and management. |
| The high ulceration risk for those who are non-compliant. |
| Subtle complications and their potential for severe outcomes. |
| How a small wound can escalate into a severe, hard-to-manage lesion. |

**13. What do you think the patient struggles to understand most about what is told to them?**

| **Responses** |
| --- |
| Daily risks. |
| The necessity of deprivation. |
| The danger of ulceration onset. |
| The time required for wound care. |
| The stricter rules they need to follow. |
| The urgency of the situation. |
| They understand a good part of it. |
| If explained correctly, no issues arise. |
| Everything. |
| The importance of being attentive. |
| Primary prevention measures like hygiene and using appropriate footwear. |
| Some patients need everything explained in great detail. |
| Terms that are too medical. |
| Advice for daily life, such as using specific footwear and foot hygiene. |
| They understand a good part of it. |
| Trusting professionals for prevention and care. |
| The timing in which ulceration can occur. |
| The actual complications, such as the significant risk posed by improper footwear. |
| They do not accept it. |
| Some patients. |
| Believing the doctor exaggerates when discussing the risk of losing a foot. |
| Care and prevention. |
| Sometimes patients are superficial in assessing the risks they face. |
| Nothing. |
| They don’t believe it could happen to them. |
| The need for appropriate footwear. |
| Patients often tire of words; empathetic communication is required. |
| The necessity of being consistent in preventive actions. |
| Certainly. |
| Why they must check their feet daily. |
| Explaining with simple, concrete terms the complications, podiatric routines, and frequency of screening tests based on risk classification. |
| The need for follow-ups and multidisciplinary controls. |
| Actions to avoid. |
| The importance of using primary prevention footwear. |
| The importance of daily care. |
| The rules to follow. |
| Protocols regarding footwear and insoles. |
| Repeating and clearly writing recommendations. |
| The risk of disease progression. |
| The likelihood of severe complications. |
| Patients often fail to understand our advice. |
| They trivialize it. |
| Most think it’s all nonsense. |
| Superficiality. |
| Few people listen. |
| The risk of ulceration. |
| I don’t know. |
| Underestimates the consequences. |
| There’s no common understanding of what should be communicated. |
| They underestimate the importance of daily care. |
| I try to put them at ease and delicately explain necessary precautions. |
| They refuse to consider the risks of amputation or life-threatening conditions. |
| The real risks, as indications are perceived as exaggerated. |
| Long-term behaviors they need to adopt. |
| Thinking the doctor exaggerates or that certain things happen only to others. |
| The risks they face. |
| The risk of complications. |
| Foot hygiene. |
| Therapeutic footwear use. |
| Orthotic therapy and the aesthetics of therapeutic footwear. |
| The importance of acute and preventive footwear phases, podiatric complications, and associated risks. |

**14. How can we help the patient?**

| **Responses** |
| --- |
| Understanding their needs and vulnerabilities. |
| Organizing ad hoc group meetings. |
| Mass communication campaigns across all media and at a national level. |
| Following them consistently. |
| Not just explaining potential complications but educating both the patient and their families. |
| Through dialogue and understanding. |
| Promoting prevention. |
| Educating family members as well. |
| Conducting screenings. |
| Following them regularly and scheduling visits. |
| Reinforcing preventive measures at every consultation. |
| Explaining the disease and its implications. |
| Disseminating information through all possible means, including informational meetings. |
| Explaining risks and seeing them more frequently. |
| Using less technical language and explaining mechanisms clearly. |
| Emphasizing the role of the caregiver in daily management and showing them how to act in the patient’s everyday life. |
| Providing proper education for both patients and caregivers. |
| Organizing group therapy sessions with psychologists. |
| Informing patients at the time of diagnosis, not when complications are already present. |
| Helping them understand prevention. |
| Creating a podiatry website with images of diabetic foot complications. |
| Providing clear and simple information. |
| Encouraging periodic reviews and monitoring by family members. |
| Offering practical examples. |
| Simplifying education to focus on key concepts initially and elaborating later. |
| Ensuring consistent communication with caregivers. |
| Using international guidelines to provide structured education and self-management promotion. |
| Insisting on recommendations and maintaining connections with foot clinics. |
| Developing educational programs even before complications arise. |
| Providing visual aids and illustrated brochures to explain risks and prevention routines. |
| Improving therapeutic communication and education. |
| Maintaining continuous education and follow-ups. |

**16. What do you think are the limitations in communication with other professionals in the multidisciplinary diabetic foot team?**

| **Responses** |
| --- |
| Time and dedicated modalities. |
| Limited understanding of the problem and each professional’s competencies. |
| Lack of cooperation and pride. |
| Limited expertise. |
| Not knowing the competencies of other professionals. |
| Sharing experiences. |
| Podiatrists often not included in multidisciplinary teams as their role is not considered important. |
| Lack of communication. |
| Incomprehensible medical language. |
| Nothing. |
| Lack of consideration for the role of the podiatrist. |
| Absence of collaboration. |
| Underestimating the contribution of each team member; GPs not referring patients to the relevant specialist. |
| Lack of understanding of the podiatrist’s role. |
| I don’t know, as I’ve never been part of such a team. |
| Communication issues. |
| Lack of collaboration spirit. |
| Prevention. |
| Variability in approaches. |
| Difficulty finding doctors who value a podiatrist's advice as they view them as inferior. |
| I don’t see any limitations. |
| No team mindset. |
| Patient management. |
| Limited collaboration among professionals. |
| Failure to recognize one's role. |
| Overwhelming workloads make communication challenging. |
| Lack of a team itself. |
| Unnecessary rivalries among colleagues. |
| Siloed workflows (PDTA), compounded by indifference or hostility to maintain power at the expense of patient outcomes. |
| Lack of podiatrists in public healthcare facilities. |
| Taking things for granted. |
| I am not part of a team. |
| None. |
| Understanding the importance of all professions. |
| Recognizing the importance of the podiatrist’s role. |
| Most hospitals lack a dedicated team. |
| Some professions undervalued by others. |
| Limited willingness to listen. |
| Continuous education and valuing each profession by accepting limitations and emphasizing competencies. |
| Communication among colleagues. |
| No experience in a diabetic foot team. |
| Limited time availability. |
| Lack of specific roles. |
| Challenges in communication in some contexts. |
| I don’t know. |
| Lack of understanding of each professional's role. |
| Handover issues. |
| Use of different terminologies. |
| Need for integrated health records across private centers. |
| Lack of spaces to share acquired knowledge. |
| No time allocated due to heavy workloads. |
| Hospitals not creating conducive environments. |
| Shared knowledge and ego management/common vision. |
| Time constraints and communication challenges. |
| Divergent perspectives and priorities. |
| Different views between internists and outpatient specialists. |
| No issues. |

**17. What about communication with those outside the TEAM?**

| **Responses** |
| --- |
| Cultural sharing on the topic. |
| Limited knowledge of the subject. |
| Incompetence and ignorance on the matter. |
| Inexperience. |
| Lack of simple and precise language. |
| Information exchange. |
| Arrogance. |
| Underestimating the disease. |
| Presumption. |
| Lack of collaboration and knowledge. |
| Failure to understand the physical and psychological impact of the condition. |
| Lack of awareness of team protocols (preventive and therapeutic). |
| Absence of connection between key professionals (e.g., podiatrist and diabetologist). |
| Communication issues. |
| Lack of awareness about the podiatry profession. |
| Time constraints. |
| Lack of understanding about diabetic foot complications. |
| Disinterest in prevention. |
| Highlighting the importance of interfacing between different professionals. |
| Limited mutual knowledge. |
| Time pressures affect the quality of communication. |
| Indifference or hostility due to power dynamics. |
| Need for more communication. |
| Team implies collaboration, sharing each professional’s knowledge without power hierarchies. |
| Some GPs are dismissive of podiatrists’ inputs. |
| Competence and qualification criteria for participation. |
| Lack of proper recognition for podiatrists’ contributions. |
| Disinterest. |
| Educating others about the syndrome's complications. |
| Difficulty communicating. |
| Resistance and hesitation in acknowledging risks. |
| Need for education on diabetic foot management. |
