# Supplementary material for: Diabetic Foot Talk-Time: framework for effective communication in diabetic foot management
Source: Front Clin Diabetes Healthc. 2025 Jun 23;6:1590570. doi: 10.3389/fcdhc.2025.1590570 (PMC12232915; doi:10.3389/fcdhc.2025.1590570)
Supplement: Supplementary file 3 [file Table3.docx]

**Meaning Units and Grouping by Similar Themes from the Responses to the Health Professionals' Questionnaire**

**11. What do you think are the main obstacles to communication with the patient?**

| **Meaning Units** | **Similar Themes** |
| --- | --- |
| **Underestimation and misperception of the disease and related risks** | - The patient does not realize how insidious and dangerous their disease is. |
|  | - Diabetes is always underestimated by patients, and they do not think at all about the consequences. |
|  | - Superficiality. |
|  | - Underestimation of the dangers they might face. |
|  | - Tendency to underestimate the disease. |
|  | - Underestimation of the problem. |
|  | - Lack of awareness of the risks they face until they experience ulcers or other serious problems. |
|  | - They pretend not to see or hear anything. |
|  | - The very topic, that is, talking about risks they do not perceive, such as inappropriate tight shoes or prolonged foot baths. |
|  | - Lack of attention to the problem. |
|  | - People who do not understand the importance of foot problems and complications even when explained, and do not perceive the problem. |
|  | - The underestimation of diabetes, an aspect I often notice during visits. |
|  | - Understanding the real risk. |
|  | - Their conventions, their culture, even regarding the use of shoes. |
|  | - Not considering diabetes as a disease. |
|  | - Not taking seriously the risks and complications that may arise over time. |
|  | - Resistance and underestimation. |
|  | - Difficulty in understanding the risks of the disease. |
|  | - The belief that diabetes is not such a serious condition. |
|  | - Little awareness of the severity of the disease. |
|  | - Poor knowledge of the problem. |
|  | - Poor knowledge of the disease. |
|  | - Understanding. |
|  | - Lack of understanding. |
|  | - Ignorance. |
|  | - Ignorance about the subject. |
|  | - The patient's lack of awareness. |
|  | - Lack of understanding of the complexity of their disease. |
|  | - Patients are not always fully aware of the complications they may face. |
|  | - The complication that may arise. |
| **Educational level and socio-economic-cultural barriers** | - Socio-cultural level. |
|  | - Social class. |
|  | - Language. |
|  | - Poor cultural education. |
|  | - Social class. |
|  | - The level of education of the patient. |
|  | - The cultural level of the patient. |
|  | - Schooling and cultural level. |
|  | - Social factor. |
|  | - Comprehension capacity, level of education, cultural, social, and cognitive factors. |
|  | - Educational level. |
|  | - Social class. |
|  | - Social class and educational level. |
| **Rejection of the clinical condition** | - Acceptance of the disease. |
|  | - Non-acceptance of the disease. |
|  | - Most patients do not accept the disease and consequently underestimate the possible damage caused by it. |
|  | - Not accepting their condition, creating a psychological myth about everything that follows. |
|  | - Indifference to the problem. |
|  | - Acceptance of the disease. |
|  | - Often dealing with patients who have not accepted the chronic nature of diabetes and struggle with prevention. |
|  | - The fact that they do not accept their condition. |
|  | - Acceptance of the condition. |
|  | - Resistance. |
|  | - Non-acceptance of the chronic condition, thus not accepting possible complications. |
|  | - Rejection of the condition. |
|  | - Rejection of the condition. |
| **Ineffective communication** | - Incorrect communication. |
|  | - Not being understood. |
|  | - Communication. |
|  | - Communication. |
|  | - Lack of information and prevention. |
|  | - Lack of adequate communication techniques. |
|  | - Empathy. |
| **Low compliance** | - Patient's disinterest. |
|  | - Low compliance of the patient. |
|  | - Resistance to treatment from patients tired of dealing with such an invasive disease. |
|  | - Attention. |
|  | - Patient compliance. |
|  | - Bad habits. |
| **Emotional factors (fear)** | - Fear. |
|  | - Anxiety. |
|  | - Feeling judged. |
| **Absence of a caregiver** | - Lack of a caregiver. |
|  | - Elderly people left alone without help. |
|  | - Absence of a caregiver for support. |
| **Limited time** | - Limited time available. |
|  | - Often little time available for consultations. |
| **Absence of a team** | - Poor cooperation from other professionals. |
|  | - Shared vision among operators. |
| **Economic resources** | - Limited financial resources. |
| **Age of the patient** | - Age. |

**12. What do you think the person with diabetic foot does not understand most about their condition?**

| **Meaning Units** | **Similar Themes** |
| --- | --- |
| **Complications and Risks** | - Further complications. |
|  | - They do not realize the severity of the lesions on their foot. |
|  | - The complications. |
|  | - The risks. |
|  | - Ulcerative risk. |
|  | - Neuropathy: mechanisms and causes. |
|  | - The numerous risks they are exposed to (e.g., a simple incorrect toenail cut). |
|  | - Underestimating their condition. |
|  | - The seriousness. |
|  | - The danger of injury. |
|  | - The severity of the risk they face. |
|  | - How easily complications can occur. |
|  | - The lack of pain leads to little attention. |
|  | - The progressive nature of the condition. |
|  | - The complications they may encounter. |
|  | - That diabetes is a chronic disease, and once complications occur, it is too late; they don’t understand the need for prevention. |
|  | - Underestimating the risks associated with diabetes. |
|  | - The seriousness of the complications they may face. |
|  | - Ulcerative risk linked to inappropriate footwear. |
|  | - The severity of the irreversible consequences of this condition. |
|  | - The real dangers. |
|  | - Problems related to altered sensitivity. |
|  | - The damage they may face. |
|  | - The risk of ulcers. |
|  | - The risks of worsening progression. |
|  | - How quickly a small lesion, if not well treated, can escalate to a more severe and hard-to-manage wound. |
|  | - Subtle complications. |
|  | - Amputation risk/awareness. |
|  | - The risks they face and the importance of daily disease management. |
| **Prevention** | - Prevention. |
|  | - The importance of appropriate footwear. |
|  | - The rules to follow. |
|  | - Importance of proper nutrition and suitable footwear. |
|  | - Prevention of the condition, which can lead to sudden changes if not managed. |
|  | - Rejection of preventive measures. |
|  | - Blood sugar control. |
|  | - Proper foot support and appropriate footwear. |
|  | - The true importance of glycemic control and the correct choice of footwear. |
|  | - The need for prevention. |
|  | - The possibility of improvement. |
|  | - Often, a lack of **information**. |
| **Severity and Complexity of the Condition** | - The complexity of the disease. |
|  | - They do not understand how a "silent" disease can cause so much damage. |
|  | - The severity of the problem. |
|  | - Their current condition. |
|  | - The general severity of their disease. |
|  | - The seriousness of the situation. |
|  | - They insist on living as if the disease does not exist. |
|  | - That diabetes is a subtle disease. |
|  | - How much their quality of life depends on their choices. |
|  | - The importance of consistent therapy and that treatment often requires time and patience. |

**13. What do you think the person with diabetic foot does not understand most about what is told to them?**

| **Meaning Units** | **Similar Themes** |
| --- | --- |
| **Prevention** | - Care and prevention. |
|  | - Trusting prevention and care professionals. |
|  | - The need for consistency in preventive measures. |
|  | - The rules to follow. |
|  | - Repeating recommendations and providing clear written instructions. |
| **Podo Daily Routine Subgroup** | - Primary prevention measures such as hygiene management and appropriate footwear use. |
|  | - Advice for daily life, including specific footwear and foot hygiene. |
|  | - The importance of daily foot checks. |
|  | - Explaining in simple, concrete terms the complications of the condition, the podiatric routine to follow, and the periodicity of screening tests based on risk classification. |
|  | - The importance of daily care. |
|  | - Underestimation of the importance of daily care. |
|  | - Foot hygiene. |
| **Offload Subgroup** | - Using suitable footwear. |
|  | - The importance of primary prevention footwear. |
|  | - Protocols for shoes and orthotic insoles. |
|  | - Therapeutic footwear use. |
|  | - Orthotic therapy and the aesthetics of therapeutic footwear. |
|  | - The importance of acute phase and preventive footwear, podiatric complications, and associated risks. |
| **Risks and Complications** | - Daily risks. |
|  | - The danger of ulcer formation. |
|  | - The severity of foot lesions. |
|  | - How inappropriate footwear can represent a significant risk. |
|  | - Sometimes they superficially assess the risks they face. |
|  | - The risk of negative disease progression. |
|  | - The real likelihood of severe complications. |
|  | - Ulceration risk. |
|  | - Underestimating the consequences. |
|  | - Not contemplating the risks of amputation or life-threatening situations. |
|  | - The real risks, as they perceive recommendations as exaggerated. |
|  | - The risks and complications they face. |

**14. How can we help them?**

| **Meaning Units** | **Similar Themes** |
| --- | --- |
| **Effective Communication** | - Through dialogue and understanding. |
|  | - Explaining the disease clearly and simply, using practical examples. |
|  | - Avoiding technical jargon. |
|  | - Making them feel understood and supported, emphasizing their role in their life story rather than labeling them solely as a "patient." |
|  | - Maintaining effective, ongoing communication. |
|  | - Being patient and available to address their needs and vulnerabilities. |
| **Structured Therapeutic Education** | - Conducting targeted group or individual sessions. |
|  | - Educating the patient and their family members about prevention and disease management. |
|  | - Using practical materials like illustrated guides or brochures. |
|  | - Simplifying education by focusing on key concepts over time. |
|  | - Insisting on recommendations and maintaining follow-up with foot clinics. |
|  | - Structured education programs before complications arise. |
| **Continuous Follow-Up** | - Regular follow-ups and monitoring. |
|  | - Encouraging periodic visits and ensuring they feel supported. |
| **Widespread Awareness** | - Mass communication campaigns on all platforms. |
|  | - Creating podiatry-focused websites and resources to raise awareness. |
| **Prevention** | - Consistently emphasizing screening and preventive measures. |
| **Caregiver Involvement** | - Educating and involving caregivers in the patient’s daily care. |
| **Team Integration** | - Establishing multidisciplinary networks and integrating specialized roles into care pathways. |

**16. What do you think is a limitation in communication with other professionals in the interdisciplinary diabetic foot team?**

| **Meaning Units** | **Similar Themes** |
| --- | --- |
| **Insufficient Collaboration and Structured Communication** | - Lack of cooperation and professional pride. |
|  | - Limited sharing of experiences. |
|  | - Podiatrists are often excluded from multidisciplinary teams as they are not considered essential. |
|  | - Lack of communication. |
|  | - Unintelligible medical language. |
|  | - Lack of respect for the role of podiatrists. |
|  | - Absence of collaboration. |
|  | - Tendency to undervalue the work of each team member, such as GPs delaying referrals to specialists. |
|  | - Lack of team spirit. |
|  | - Difficulties in accepting advice from podiatrists, viewed as inferior. |
|  | - No concept of teamwork. |
|  | - Poor collaboration among different professionals. |
|  | - Overloaded work schedules making communication challenging. |
|  | - Non-existence of formal teams in most hospitals. |
|  | - Unnecessary rivalry among colleagues. |
|  | - Dividing the care pathway into silos, often combined with indifference or hostility to maintain control at the expense of patient outcomes. |
|  | - Lack of established teams in many hospitals. |
|  | - Limited willingness to listen. |
|  | - Difficulties in communication at certain points. |
|  | - Hand-off processes between professionals. |
|  | - Use of different terminologies. |
|  | - Shared knowledge, ego management, and a common vision. |
|  | - Communication breakdowns. |
|  | - Not sharing decisions or consulting other team members. |
|  | - Lack of communication and respect for roles. |
| **Lack of Awareness of the Problem and Roles** | - Limited knowledge of the issue and the competencies of each professional. |
|  | - Not understanding the specific skills of other professionals. |
|  | - Lack of understanding of the podiatrist's role. |
|  | - Variability in approach among professionals. |
|  | - Failure to recognize one's own role. |
|  | - Lack of appreciation for the importance of all professions. |
|  | - Not recognizing the significance of the podiatrist's role. |
|  | - Some professions undervalued by others. |
|  | - Lack of specialized roles. |
|  | - Lack of understanding of the role of each professional. |
| **Time Constraints** | - Limited dedicated time and resources. |
|  | - Insufficient time for effective collaboration. |
|  | - Lack of time to provide detailed reports to colleagues. |
|  | - Limited time for dialogue among professionals. |
| **Different Perspectives** | - Different viewpoints and priorities. |
|  | - Diverging perspectives between internal medicine specialists and outpatient professionals. |
|  | - Different perspectives among team members. |
| **Lack of Shared Spaces for Information Exchange** | - Absence of environments to share information acquired in individual practices. |
|  | - Hospitals not creating conducive spaces or conditions for professional collaboration. |

**17. And with those who are not part of the TEAM?**

| **Meaning Units** | **Similar Themes** |
| --- | --- |
| **Lack of Knowledge and Contextual Differences** | - Limited knowledge of the subject. |
|  | - Incompetence and ignorance about the topic. |
|  | - Inexperience. |
|  | - Arrogance. |
|  | - Underestimation of the disease. |
|  | - Presumption. |
|  | - Lack of understanding of the psychological and physical impact of the pathology. |
|  | - Unawareness of team protocols (preventive and therapeutic). |
|  | - Lack of understanding of diabetic foot complications. |
|  | - Lack of mutual knowledge. |
|  | - Disinterest. |
|  | - Spreading awareness about the syndrome's complications. |
|  | - Formation is needed before creating a team. |
|  | - Resistance to understanding diabetic foot risks. |
|  | - Lack of understanding of the patient's real condition and risks due to inexperience. |
|  | - Lack of interest in prevention. |
| **Reduced Awareness of the Team & Professional Silos** | - Absence of networks between key professionals, such as podiatrists and diabetologists. |
|  | - Lack of knowledge about the podiatry profession. |
|  | - Difficulty finding doctors who value podiatric advice. |
|  | - Poor representation of podiatrists in public facilities. |
|  | - General practitioners often dismiss podiatric concerns or requests for collaboration. |
|  | - First, document the podiatrist’s competencies. |
|  | - Lack of integration of podiatrists. |
|  | - Limited consideration for other professions due to lack of awareness. |
| **Poor Interprofessional Communication and Collaboration** | - Failure to speak simply and clearly. |
|  | - Insufficient exchange of information. |
|  | - Cultural sharing on the topic is missing. |
|  | - Lack of collaboration and knowledge sharing. |
|  | - The communication process. |
|  | - Demonstrating the importance of interfacing with different professionals. |
|  | - Tools and time limitations hinder communication. |
|  | - Indifference or hostility due to fears of losing control. |
|  | - More communication is needed. |
|  | - Teams must collaborate without hierarchical barriers. |
|  | - Challenges in communication among colleagues. |
|  | - Difficulty understanding technical language. |
|  | - Missed opportunities for professional interaction. |
|  | - Lack of cooperation in shared facilities. |
| **Lack of Time** | - Scheduling issues. |
|  | - Limited time for collaboration. |
|  | - Difficulties in meeting due to busy schedules. |
|  | - Lack of platforms for professionals to share information. |
